# Methods for the establishment of enzymatic mechanisms – from QM to ML

**DOI:** 10.1039/d6sc02180h

**Published:** 2026-07-22

**Authors:** Rui P. P. Neves, João T. S. Coimbra, Pedro Paiva, Umberto Raucci, Sudip Das, Ana R. Calixto, António J. M. Ribeiro, João P. M. Sousa, Pedro Ferreira, Enrico Trizio, Pedro A. Fernandes, Michele Parrinello, Maria J. Ramos

**Affiliations:** a LAQV, REQUIMTE, Departamento de Química e Bioquímica, Faculdade de Ciências, Universidade do Porto Rua do Campo Alegre s/n Porto 4169-007 Portugal mjramos@fc.up.pt; b Italian Institute of Technology Genova GE 16163 Italy michele.parrinello@iit.it; c LAQV, REQUIMTE, Laboratório de Química Orgânica e Farmacêutica, Departamento de Ciências Químicas, Faculdade de Farmácia, Universidade do Porto Rua de Jorge Viterbo Ferreira Porto 4050-313 Portugal

## Abstract

We offer a practical and conceptual introduction to some of the current approaches to modelling enzymatic reaction mechanisms, ranging from quantum mechanics (QM), molecular mechanics (MM), and hybrid QM/MM approaches to enhanced sampling methods, knowledge-based approaches, and machine learning (ML) advances. We discuss how static and dynamic QM/MM approaches, as well as multi-PES strategies, have contributed to understanding the role of conformational diversity, electrostatic preorganization and solvent participation in the determination of catalytic barriers and reaction paths. We focus on how advanced sampling techniques and data-driven collective variables have enabled the exploration of rare events and reaction coordinates, as well as how knowledge- and rule-based approaches have facilitated the interpretation and hypothesis generation for different families of enzymes. Recent developments in ML potentials, ML collective variables, and committor-based sampling are presented as innovative methods that have been able to address some of the current challenges in accuracy, sampling efficiency, and the identification of low-dimensional representations of reaction coordinates. A case study of α-amylase demonstrates how the combination of these strategies leads to a comprehensive understanding of enzyme reactivity, from the chemical to the conformational level. Collectively, these developments contribute to a predictive understanding of enzymatic catalysis, which will have extensive implications in enzyme engineering, sustainable chemistry, and drug discovery. Advances in high performance computing, automated simulation pipelines and data formats will likely make multiscale simulation more accessible and reproducible. Simultaneously, the combined application of mechanistic knowledge, ML, and experimental validation will hopefully advance the discovery and optimization of biocatalysts with well-defined properties, tailored to meet pressing societal needs, such as plastic biodegradation, carbon sequestration, sustainable synthesis and personalised medicine.

## Abstract 2

We review state-of-the-art computational modelling of enzyme reaction mechanisms, highlighting hybrid quantum mechanics/molecular mechanics (QM/MM) methods and the emerging role of machine learning in the field.

## Graphical abstract

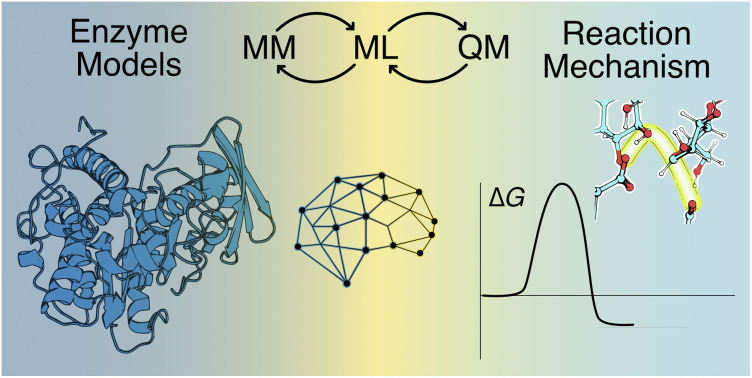

## 1 Introduction

Enzymes are nature's highly efficient, specific, and tunable catalysts, capable of performing complex chemical transformations under mild conditions with extraordinary selectivity. Their applications span broad scientific domains: treating diseases,^1^ addressing complex environmental problems,^2,3^ and improving sustainability in industrial ecosystems.^4,5^ Their versatility stems from the wide range of reactions catalysed by enzymes, frequently under diverse conditions, enabled by their molecular complexity and structural flexibility.

**Fig. 1:**
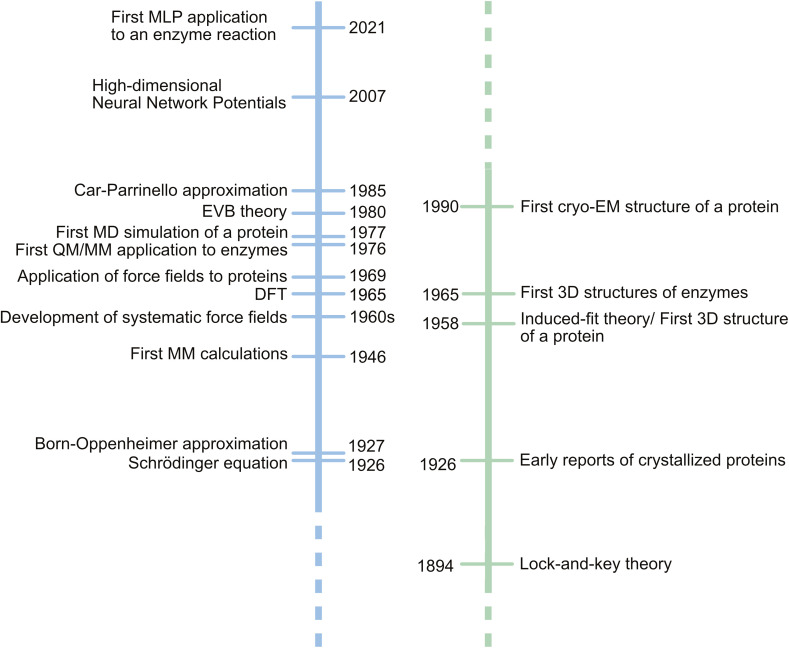
Early events in computational chemistry and structural biology applied to proteins. Both fields have been recognized with numerous Nobel Prizes, including the 1933 Physics Prize awarded to Schrödinger and Dirac and the Chemistry Prizes awarded to Pauling (1954), Kendrew and Perutz (1962), Hodgkin (1964), Anfinsen (1972), Fukui and Hoffmann (1981), Michel, Deisenhofer and Huber (1988), Marcus (1992), Kohn and Pople (1998), Wüthrich (2002), Karplus, Levitt and Warshel (2013), Dubochet, Frank and Henderson (2017), and Baker, Hassabis and Jumper (2024). This list is not exhaustive but highlights pivotal contributions bridging theory and structural determination.

Enzyme modelling addresses the understanding of how the linear sequence of amino acid residues folds into a three-dimensional (3D) structure and how this arrangement drives chemical reactivity, capturing the sequence–structure–function relationship. Its development is tightly linked to the advances in two complementary fields: computational chemistry and structural biology. Each field has progressed *via* paradigm-shifting milestones (Fig. 1).

One of the earliest examples is that enzymes were initially regarded as biological catalysts of unknown structure^6^ until Sumner first crystallized urease,^7^ sparking the field of structural biology. Later, in the mid-20th century, Koshland proposed his view of substrate binding to an active site in the context of protein synthesis,^8^ establishing the foundation of the “induced-fit” theory, which expanded Fischer's static “lock-and-key” model.^9^ Koshland hypothesized that enzymes adjust to their substrate and *vice versa*, analogous to the way a glove fits a hand,^10^ an idea that initially met resistance.^11^ Less than a decade later, Koshland's hypothesis was validated with the first atomic resolution crystal structure of hen egg white lysozyme, showing not only substrate distortion but also slight differences in active site residues between the substrate-free and substrate-bound enzymes.^12,13^

Alongside the evolution of structural biology, computational chemistry has developed rapidly over the last 50 years, fuelled by both algorithmic advances and increases in computational power, which enabled the modelling of increasingly complex enzyme systems and their dynamic behaviour. Within computational chemistry, two classes of methods evolved in parallel during this period: molecular mechanics (MM) and quantum mechanics (QM).

MM methods can be traced back to the 1940s with conformer exploration of diphenyl derivatives through manual calculations.^14^ Around the same time, Hill proposed a potential energy function, commonly known as a force field, whose mathematical terms are still in use today.^15^ During the 1960s, several force fields were developed for different types of biomolecules, in which atoms and bonds are treated as spheres connected by springs.^16–18^ Force fields employed empirical parameters including equilibrium values and force constants for bonds, angles, and dihedrals, as well as non-bonded parameters such as van der Waals radii and atomic charges, designed to reproduce biomolecular behaviour, culminating in their application for refinement of X-ray structures.^19,20^

The integration of Newton's equations of motion with forces computed from a force field led to one of the landmark achievements in computational chemistry, known as molecular dynamics (MD). The first MD simulation of a biological macromolecule, the bovine pancreatic trypsin inhibitor (BPTI), lasted only 9.2 ps in a vacuum, but it already revealed the flexible nature of proteins, demonstrating that proteins exist as ensembles of fluctuating conformations rather than single static structures.^21^ Nowadays, computing power has advanced so rapidly that it is possible to simulate large biomolecular assemblies containing millions of atoms for hundreds of nanoseconds to microseconds (or even milliseconds), using state-of-the-art high performance computing (HPC) and graphical processing units (GPUs).^22,23^ More details on MM's rapid development can be found in dedicated reviews.^16,24–28^

Biochemical processes range from large and slow molecular motions, such as domain and loop rearrangements (10^−6^ to 10^−9^ s), to faster bond vibrations and bond-breaking/formation events, in the 10^−12^ to 10^−15^ s range.^29,30^ While traditional MM methods can capture the former, they fail to model the latter as they cannot account for breaking and formation of bonds. Consequently, accurate modelling of enzymatic reactivity requires electronic structure methods, a branch of QM dedicated to characterizing chemical systems from their electronic wavefunction or electron density.

The development of these methods owes its success to two pivotal advancements in the late 1920s: the formulation of the Schrödinger equation,^31^ which enabled the description of systems from first principles, and the Born–Oppenheimer approximation.^32^ The latter simplified QM by decoupling nuclear and electronic motions, an approximation made possible due to their large difference in mass (*m*_proton_ = 1836 atomic units *vs. m*_electron_ = 1 atomic unit) and velocity. Although these theories were first applied by Heitler and London to the H_2_ molecule to describe the bond between hydrogen atoms,^33^ explicitly accounting for electron–electron interactions in larger molecules was far too complex at the time. It was not until the advent of computers in the 1950s that these complex interactions could be treated computationally.

After the initial developments, electronic structure methods branched into three main classes: (i) wave function theory (WFT) directly derived from fundamental quantum physics, offering the most precise yet the most computationally expensive approaches; (ii) density functional theory (DFT) which, instead of using the full many-electron wave function, employs the simpler concept of electron density, *i.e.*, the probability density of finding any of the system electrons at a given point in space, to determine system energy and other electronic properties; (iii) semiempirical (SE) methods, which incorporate empirical parameters fitted to experimental data^34^ and include several approximations, including neglect of overlap between different atomic orbitals,^35–39^ permitting treatment of entire enzyme active sites but at the expense of limited chemical accuracy outside systems or reaction types not represented in the parameterization sets. QM-based methods are currently in a mature stage of development and have been extensively discussed in the literature.^40–42^ More recently, ML-trained potentials are being developed to improve the scalability of computations while minimising trade-offs in accuracy.

Enzyme modelling leverages both QM and MM methods to capture fast chemical events and slower conformational dynamics, respectively. The need to accurately describe both types of motions in a single framework motivated the development of hybrid QM/MM approaches. In their seminal QM/MM work, Warshel and Levitt investigated the glycosidic bond cleavage in hen egg white lysozyme, which had previously solved crystal structures, supporting the validity of their simulations. Their work showed that the electrostatic stabilization of the enzyme's active site (described at the SE level), by the enzyme body and solvent, enhances the rate of carbocation formation, providing a more realistic description of the lysozyme reaction mechanism and the resulting free energies.^43^

As mentioned earlier, computational chemistry progressed through insights from experiments, but innovative theoretical models have likewise motivated experiments designed to test their predictions. Nowadays, this loop between simulations and experiments is indispensable for an in-depth understanding of enzyme reaction mechanisms, frequently complementing experimental work. Agreement between simulation and experiment has been reported in multiple studies, including the occurrence of alternate reaction geometries for the thiol/disulfide equilibrium in thioredoxin,^44,45^ the observation of a direct displacement mechanism in type I/II l-asparaginases, instead of a ping-pong mechanism, subsequently validated by experiments,^46,47^ the accurate estimation of the near-attack conformation and transition state (TS) free energies of chorismate mutase,^48,49^ the characterization of elusive reactive intermediates in cytochrome P450,^50–52^ and countless other examples.^53,54^

The breakthrough of QM/MM lies in combining the strengths of both QM and MM. The QM treatment of the enzyme active site enables the simulation of bond-breaking and bond-forming events, while the MM layer captures the structural and electrostatic influence of the enzyme scaffold on reactivity.

Earlier QM/MM studies often relied on a single static enzyme conformation, thereby overlooking the contribution of other conformations to the activation barrier. Since then, the field has evolved to include approaches such as multi-PES QM/MM, which enables the exploration of multiple potential energy surfaces (PESs) associated with different enzyme conformations. More exhaustive exploration of the reaction space is now performed through QM/MM MD simulations that, although limited to relatively short simulation timescales, can unveil additional details of active site dynamics when combined with enhanced sampling techniques, implicitly accounting for entropic contributions.^55^

In the current ML era, the computational chemistry community is increasingly developing ML-based models to expand the scope of enzymatic simulations, enabling access to longer timescales and larger systems than previously feasible. On one hand, ML models can be trained on quantum-mechanical reference data to predict energies and forces, enabling the construction of accurate interatomic potentials. These models provide a computationally efficient representation of reactive PESs and allow performing MD simulations at near-QM accuracy over extended time and length scales.^56^ On the other hand, ML methods can be integrated with enhanced sampling strategies to improve the exploration of rare events, such as enzymatic reactions, thereby facilitating a more comprehensive characterization of complex reaction mechanisms and revealing atomistic details that would otherwise remain difficult to access.^57^

Throughout this review, we aim to cover computational enzyme modelling in different contexts, providing a practical overview of common approaches to model reaction mechanisms, along with their strengths and pitfalls. Finally, a case study on α-amylase illustrates how different enzyme modelling approaches can be applied to unveil distinct dimensions of enzyme reactivity.

## 2 Modelling enzymatic systems

### 2.1 Computational strategies for modelling enzyme-containing systems

Capturing the biochemical behaviour of an enzyme-containing system through *in silico* methods begins with accurately modelling its structure, which is essential for any subsequent simulations. This process starts with obtaining a high-quality enzyme structure, which must be identified, thoroughly analysed, and refined to ensure its suitability for further simulations. Typically, the structures of proteins, enzymes, and other macromolecules are stored in large repositories such as the RCSB Protein Data Bank (PDB).^58^ As of 2026, the RCSB PDB holds nearly 250 000 structures, the majority of which (∼80% of all deposited structures) were determined through X-ray diffraction, with additional contributions from cryo-electron microscopy (cryo-EM) and nuclear magnetic resonance (NMR).^59^ Of these, a significant fraction contains enzymatic domains, *i.e.*, they correspond to enzyme structures. Despite this abundance, many important enzymes remain structurally unresolved.

When high-quality enzyme models are unavailable in public databases, often because X-ray diffraction or cryo-EM methods fail to resolve the electron density or the electrostatic potential map of disordered or flexible regions of proteins,^60^ computational approaches can be used to refine existing structures or predict 3D representations from sequence information. In the first scenario, if residues or segments are missing from an experimentally determined model, the incomplete enzyme can be superimposed onto a closely related homologue model containing the missing residues, and a complete model can be generated after transferring the corresponding missing atomic coordinates. Predicting the complete 3D structure of enzymes from their amino acid sequences typically requires more sophisticated computational techniques, such as homology modelling,^61,62^ protein threading (also known as fold recognition),^63^ or *ab initio* approaches.^64^

Homology modelling infers the structure of an enzyme by aligning its sequence with that of homologous enzymes that have a known structure, which serve as templates. This method leverages the fact that the 3D structure of proteins is generally more conserved than their primary amino acid sequence.^65^ When suitable templates with sufficient sequence identity are available, this approach usually yields highly accurate models at a relatively low computational cost. Protein threading, conceptually related to homology modelling, can be employed when the sequence identity between the target enzyme and all available homologues is insufficient for reliable homology modelling.^63^ Instead of relying on close homologues, threading uses a comprehensive library of known protein folds to identify the most likely structural scaffold for the input sequence. In both homology modelling and protein threading, the quality of the model heavily depends on the availability of appropriate templates or folds and on the accuracy of the sequence–structure alignment.

In contrast, *ab initio* modelling can be used when the target enzyme's sequence does not show structural similarity with any solved structure. Instead, this method is based solely on the amino acid sequence of the target enzyme, under the assumption that a protein/enzyme in its native state resides in its lowest free-energy minimum.^66^ Classical *ab initio* methods rely on physical principles and employ advanced sampling techniques to explore the conformational space, but this comes at a significant cost: these methods are much more computationally demanding and time-consuming than template-based methods. Therefore, homology modelling and threading are usually preferred when suitable structural information exists, whereas *ab initio* modelling becomes an essential tool for modelling enzymes lacking homologues or when existing enzyme structures fail to provide an adequate scaffold.

The development of ML and deep neural network-based methods, many of which are data-driven, has significantly transformed the landscape of *ab initio* prediction in recent years.^67^ These methods can achieve unprecedented levels of accuracy by combining statistical patterns learned from large protein sequence and structure databases, setting them apart from traditional physics-based *ab initio* approaches. One notable example is DeepMind's AlphaFold2,^68,69^ which has generated structural predictions for entire proteomes and showed unparalleled accuracy in the 14th Critical Assessment of Structure Prediction (CASP14) competition.^70^ It has significantly expanded researchers' capacity to tackle long-standing experimental obstacles for proteins that are very hard or even sometimes impossible to crystallize. As of 2025, the AlphaFold Protein Structure Database,^71^ jointly developed by DeepMind and EMBL-EBI, contains predictions for the proteomes of 48 distinct organisms and more than 241 million protein structures, already providing important insights into protein functions and interactions, and these numbers continue to grow. The AlphaFold2 method still has, however, some well-known limitations: it struggles to accurately predict the structure of highly dynamic enzymes (*i.e.*, those with several metastable states) from a single input sequence, it cannot model post-translational modifications, it cannot reliably predict structural changes caused by single-point mutations, it does not natively model ligand binding, and its accuracy is highly dependent on the multiple sequence alignments required, which can be very poor for enzymes with a few or no close homologues in existing databases.^68^ Additional methods that build upon AlphaFold2 have managed to circumvent some of the limitations mentioned above, including AF-Cluster,^72^ which enables AlphaFold2 to sample alternative conformational states of metamorphic proteins with high confidence. Deep-learning methods are now even more accessible and reproducible, thanks to the development of recent alternative frameworks such as RoseTTAFold,^73^ ColabFold,^74^ ESMFold,^75^ and OpenFold.^76^ More recently, AlphaFold3 ^77^ has improved predictions for flexible and challenging proteins, relying less on deep sequence alignments and more effectively capturing alternative conformations and structural variability. Altogether, these advancements in deep-learning-based *ab initio* prediction methods have transformed what was once a challenging and computationally demanding process into a practical and reliable solution in cases lacking homologous structural templates.

Another promising application of these deep-learning methods is the design of entirely new proteins, *i.e.*, enzymes that do not exist naturally.^78–80^ This unlocks new possibilities for enzyme engineering. Researchers can explore hypothetical mutations, develop enzymes with new catalytic functions, or enhance enzymes' stability and specificity, features that are highly desirable for commercial, industrial, or therapeutic applications. Moreover, because these methods produce reliable structural models, they complement experimental mutagenesis studies, contributing to enzyme engineering pipelines that are not only more efficient but also easier to understand.

In most biological processes, enzymes do not act in isolation but within complex systems that may include substrates, products, inhibitors, cofactors, metal ions, nucleic acids, and other interacting proteins. To assemble realistic computational models of such systems, it is essential to capture this complexity, as these components influence enzyme conformation, dynamics, and catalytic efficiency.^81,82^ Therefore, once a reliable enzyme structure has been obtained *via* experimental or computational sources, it is necessary to integrate it into its functional environment, which often requires further strategies to model small molecules, additional macromolecular entities, and the surrounding solvent or membrane environment. In some cases, the experimentally resolved structures comprise those components (typically inhibitors, structural metal ions, cofactors, *etc*.), often positioned near their native functional positions. Whenever that is not possible, one may again resort to structural alignment with homologous structures that contain those components and transfer their coordinates accordingly. A recent tool, AlphaFill,^83^ streamlines this process by leveraging sequence and structure alignment heuristics to transfer missing ligands, cofactors, and/or ions from experimentally determined structures to protein models. Originally developed for AlphaFold-predicted structures, AlphaFill can also work on user-provided structures, including those derived from experimental data.

Natural substrates, however, are frequently missing from existing experimental structures of enzymes,^84^ primarily because they react with the enzyme faster than the timescale required for structural determination. As a result, capturing a true enzyme–substrate complex experimentally is challenging, and computational modelling becomes crucial for reconstructing the active complex. This can involve additional modelling strategies such as coordinate transfer from homologues or tools such as AlphaFill and often requires other approaches, including molecular docking, which predicts the preferred pose of a ligand within the enzyme's binding site and provides a scoring metric correlated with binding affinity (*i.e.*, how tightly the compound binds). Typically, a docking run generates multiple enzyme–ligand poses, which are ranked by a scoring function. The top-ranked poses may serve as starting points for more detailed computational studies, such as MD simulations. Despite its widespread use, this approach is still far from perfect, mainly due to approximations, such as the use of rigid receptor models (although several docking platforms now incorporate receptor flexibility to partially mitigate this limitation), scoring inaccuracies and simplified treatment of solvation, which can compromise the reliability of the predicted binding poses. For a more comprehensive discussion on molecular docking, see a recent review by Paggi *et al.*^85^

Once again, AI-driven methods have pushed the boundaries of enzyme modelling by improving both the speed and accuracy of predictions for enzyme-containing systems. The recently released AlphaFold3 ^77^ is reported to predict complexes that include almost all types of molecules found in the PDB. Notably, it can model metal ions and metal binding sites, and it achieves high accuracy in predicting covalent modifications such as glycosylation, covalently bound ligands, and modified amino acids or nucleic acid bases. Furthermore, its developers report that it outperforms specialized tools when modelling protein–ligand and protein–nucleic acid interactions. Together with AlphaFold Server, a free, easy-to-use platform that harnesses the power of AlphaFold3, researchers worldwide can access these predictions without requiring extensive computational resources or deep-learning expertise. However, the server currently supports only a limited set of ligands, DNA/RNA, and ions, and does not allow the inclusion of custom ligands. The full capabilities of AlphaFold3 require a local installation of the software and model parameters provided by Google DeepMind. The state-of-the-art and openly accessible Chai-1 model for molecular structure prediction (also available as a web server)^86^ overcomes this limitation by allowing users to input arbitrary ligands through the ligand-specific Chemical Component Dictionary (CCD) code or a Simplified Molecular Input Line Entry System (SMILES) sequence. More recently, AlphaFold3-based models such as Boltz-2 ^87^ and OpenFold3 ^88^ have become available in web servers, democratizing access to these predictive tools for the scientific community. These deep-learning-based approaches can account for structural context and protein flexibility more effectively than classical scoring functions.

Regardless of the modelling approach, the resulting models typically require further refinement to address modelling inaccuracies, such as steric clashes, misplaced loops or non-optimal orientations of the docked/modelled ligand, which can compromise the overall quality of the model.^89^ In addition to these structural issues, an often underestimated but crucial aspect of enzyme modelling is the assignment of appropriate protonation states for ionizable residues. In enzymatic systems, protonation states strongly influence electrostatic interactions, substrate binding, and the reaction mechanism itself.^90–92^ An accurate protonation state assignment is thus essential for enzymes featuring highly coupled catalytic architectures, such as catalytic dyads or triads, or extended H-bond networks, where small changes in protonation states can significantly impact the reaction pathway. Several computational tools have been developed to assist with the assignment of protonation states and p*K*_a_ value estimation, including empirical approaches such as PROPKA,^93^ continuum electrostatics-based approaches such as PyPKA,^94^ and more recent ML-based tools such as DeepKa,^95^ highlighting the emerging role of AI-driven approaches in protonation state prediction.

Energy minimization protocols, as well as MD simulations, are frequently used to relax the system, alleviate local strain, and bring the model closer to a physically realistic state.^89,96,97^ In MD simulations, atomic motion is simulated by solving Newton's equations of motion, yielding a trajectory describing the behaviour of the enzyme-containing model over time while allowing the system to equilibrate under near-physiological conditions. Ideally, one should run multiple long-timescale MD replicate simulations to thoroughly sample the conformational space of the system and capture phenomena such as large-scale domain motions or ligand diffusion. These events occur on timescales beyond the microsecond to millisecond range, as they require crossing high-energy barriers to transition between different conformations.^98,99^ The following reviews provide a comprehensive summary of recent developments in MD and enhanced sampling methods, which can facilitate rare transitions and enable broader exploration of conformational space within workable timeframes (discussed later in Section 4.3).^100,101^

Finally, MD simulations can provide insights into catalytic pre-organisation, a topic of great interest in enzyme studies. By simulating enzyme–ligand complexes and monitoring the position of the ligand, the geometry of the active site, key interatomic distances, and noncovalent interactions, MD simulations can reveal whether a given enzyme–substrate conformation is likely to be catalytically compatible with the experimentally observed turnover.^102^ This is particularly useful for validating modelled complexes or guiding further studies for enzyme engineering or drug design. However, although classical MD alone can assess preorganisation or substrate positioning, it cannot model chemical steps. The modelling of chemical steps will be addressed in detail further ahead.

Building on these strategies, the next challenge is understanding enzymes' behaviour as they interact with much larger and more complex substrates or materials, such as polymers, nanoparticles, and biological membranes, where catalytic efficiency and specificity are largely determined by adsorption, conformational adaptation, and substrate transport.

### 2.2 Modelling enzyme–membrane environments

Biological membranes contain a chemically diverse set of lipid molecules that are present in different amounts and proportions. These structures can vary dramatically among cell types, tissues, organelles, membrane leaflets, and even membrane subdomains.^103,104^ This diversity affects the collective behaviour of lipids and determines membrane structure and properties, including fluidity, thickness, curvature, and phase separation, all of which are essential for membrane function and dynamics.^104^

There is now a growing body of evidence showing that lipids can regulate membrane-bound proteins through both specific and non-specific interactions.^105–107^ Annular lipids form a dynamic belt around membrane proteins, such as transporters, influencing their immediate environment and establishing multiple transient contacts.^108^ These protein-proximal lipids often differ from the ones in the bulk. However, bulk membrane properties, such as thickness and elasticity, can also influence the energetics and kinetics of protein conformational transitions.^108,109^ In contrast, non-annular lipids can form tight and specific contacts at defined sites within the protein structure. Examples can be found for cholesterol, cardiolipin, and phosphoinositide molecules which, for instance, have been shown to interact with high specificity and affinity with certain membrane-bound proteins.^105,108,110–113^ Non-annular lipids are essential regulators of protein activity, as they can mediate oligomerization, modulate interactions, or stabilize conformational states.^108^ Some proteins, however, can be relatively agnostic to their solvating lipids.^105^ In turn, membrane-bound proteins can distort the membrane and surrounding lipids, highlighting the bidirectional nature of protein–lipid interactions.^114,115^ Adding to this complexity, membrane-bound proteins can be regulated by partner proteins, subcellular localization, or post-translational modifications.^108^

Finally, another crucial factor influencing protein–lipid interactions is the asymmetric distribution of lipids between the two leaflets of a membrane.^105,116^ Membrane leaflets can differ not only in headgroup identity but also in acyl chain saturation. This is reflected in the asymmetry of protein transmembrane domains, which appears to be conserved across eukaryotes.^117^ Integral membrane proteins can also respond to asymmetric lipid distributions, and some studies have shown that the activity of membrane-embedded enzymes or the activation of mechanosensitive channels can be modulated by membrane asymmetry.^118^

All the factors described above highlight the importance of considering the membrane environment when studying membrane-bound proteins. An accurate computational model should ideally account for the complex interactions of membrane lipids, water, and ions, because these elements affect the protein structure, dynamics, and function. Replicating realistic membrane composition and assembly, nevertheless, remains a challenging task, and modelling membrane-associated enzymes adds another level of complexity.

Membrane proteins and their membrane environment, however, are often difficult to characterize. The number of membrane protein structures deposited in the RCSB PDB remains relatively modest, largely due to technical challenges related to their expression, purification, and structure determination.^119,120^ Moreover, the native membrane environment is often not captured in these structures. These caveats also complicate quantitative biophysical characterization of protein–lipid interactions, including identification of specifically bound lipids and binding affinities.^120^

Recent advances in structural and functional methods, such as nanodisc technology,^121^ solid-state NMR,^122^ cryo-EM,^123–126^ and AI-based protein structure prediction methods (*e.g.*, AlphaFold),^127,128^ have nonetheless provided important insights into the architecture of membrane proteins, as well as their immediate lipid environment.^129^ Methods like mass spectrometry (MS) have also been crucial for identifying and quantifying native lipid species tightly associated with membrane proteins.^130^ Furthermore, the development of fluorescence techniques has transformed membrane biophysical studies.^131^ Techniques such as fluorescence recovery after photobleaching (FRAP), Förster resonance energy transfer (FRET), fluorescence correlation spectroscopy (FCS), or the combination of FCS with super-resolution microscopy (STED-FCS) have enabled the real-time study of dynamic phenomena in living cell membranes with high temporal and spatial resolution.^132–136^

In parallel, computational approaches, particularly MD simulations, have been instrumental in revealing the dynamic behaviour of these proteins within membranes.^119,120^ The development of computational methods, particularly coarse-grained (CG) force fields, has enabled the simulation of more complex and realistic membrane environments.^137^ In CG simulations, the complexity of molecular systems is reduced by grouping atoms into larger CG units or beads. This significantly decreases the number of particles to track, improving the computational efficiency and enabling the study of larger systems and longer timescales that are typically inaccessible with atomistic simulations.^138–141^ Computational studies have been further driven by rapid advances in hardware, making it possible to simulate increasingly large systems over timescales that are biologically relevant for membrane protein dynamics. More examples of realistic membrane simulations are emerging rapidly in the literature,^137,142–145^ supported by experimental advances that offer more accurate models for computational procedures. Computation, in turn, provides a dynamic and molecular view of membrane behaviour that is often difficult to capture experimentally.^137^

On a different scale, to study chemical reactivity in membrane-associated enzymes, we typically rely on hybrid methods, where the active site is treated with QM, while the remainder of the protein environment is described using MM (this will be reviewed later). A solvation sphere or water box is usually added to mimic the bulk aqueous environment. In practice, conformational sampling is often first performed using larger models that include the membrane environment, whereas the subsequent QM/MM description of the chemical step typically relies on smaller truncated models. Historically, the lipid environment has often been excluded from QM/MM models due to the significant computational cost involved; properly accounting for these environmental effects may generate large simulation systems of up to a few million atoms.^146^ As a result, the problem is often approached in two stages: (i) sampling the conformational space of the enzyme–substrate complex using models, which may or may not include the membrane environment; (ii) studying the reaction mechanism itself using a truncated, smaller model where only the active site and its immediate surroundings are considered. This strategy allows for the exploration of catalytically relevant conformations using low-resolution models (such as MM), followed by high-resolution or multi-resolution QM/MM methods to study reaction mechanisms more accurately. This approach is often described as multiscale modelling, where multiple simulation methods are used to study changes that occur at various spatio-temporal scales and how these could be linked or coupled.^147^

More recently, computational studies have emerged that consider the full enzyme–substrate–membrane complex in multi-resolution simulations. Some of these studies report that the membrane environment has a negligible impact on the catalysed reaction energetics,^148^ though this could be very case specific. In addition, the problem should not be viewed purely from a chemical point of view. Substrate and enzyme binding dynamics and product release are also crucial components of enzymatic catalysis.^148–153^ In this context, the presence of a lipid environment becomes highly relevant, as it can influence diffusion rates, drive allosteric activation of membrane proteins, and modulate the conformational dynamics of these enzymes. For example, in phospholipase A_2_ (PLA_2_), the presence of the membrane increases the reaction rate by several orders of magnitude.^154^

Modelling membrane composition is also a relevant challenge. Due to limited information about lipid composition and its functional relevance, researchers often rely on simplified mono-lipid models that approximate the general physicochemical characteristics of different biological membranes. This holds for both experimental and computational research.^155^ For example, zwitterionic lipids are typically used when studying eukaryotic membranes, while negatively charged phospholipids are employed to mimic bacterial membranes. This is a major oversimplification, and a more realistic approach would involve simulating diverse lipid mixtures, crowded environments (membrane proteins can contribute up to 25–75% of the membrane mass),^156^ or even accounting for chemical or potential gradients across the membrane, although such simulations remain computationally demanding and often rely on coarse-grained or multiscale approaches.^114^ Building such complex systems is, of course, nontrivial. Here, platforms like CHARMM-GUI Membrane Builder^157^ let researchers design mixed lipid bilayers with precise composition and automate system setup for simulations. Importantly, they also support the inclusion of proteins, ions, or ligands, making them an important tool for modelling multicomponent membrane environments. More recently, tools like TS2CG,^158^ or LipidWrapper,^159^ can help build stable models of large-scale membranes/systems that contain complex curvature and diverse lipid and protein compositions.^119^

However, realistic membrane composition also adds a significant computational cost, because lipid diffusion is slow, and therefore longer simulations are required to properly equilibrate these systems. In this context, CG simulation methods are essential. By reducing the number of degrees of freedom, they enable simulations of larger systems over much longer timescales. These extended timescales are critical for proper membrane equilibration and for capturing the slower dynamics of lipids and protein–lipid interactions. Atomistic resolution can then be recovered through backmapping,^160^ allowing for more detailed analysis of specific interactions and the calculation of precise structural or energetic properties. Popular CG methods include physics-based models, such as the well-known MARTINI force field,^161–163^ widely used for simulating a broad variety of (bio)molecular systems, and structure-based protein models, also called Gō-type models,^164–166^ which favour native contacts to efficiently portray folding and assembly events. Other popular CG approaches have been extensively reviewed elsewhere.^138,141,167^

In conclusion, recent developments in structural resolution methods, modelling techniques, computational resources, and AI-based protocols are significantly advancing membrane enzymology studies. It is increasingly clear that considering the full complexity of biological membranes is essential for capturing the structural and functional aspects of membrane-bound enzymes. Naturally, increased model complexity often requires longer simulation times to properly sample lipid–protein dynamics and interactions. In this context, multiscale modelling emerges as a crucial tool for deciphering the reaction mechanisms of these enzymes that play a key role in various cellular processes. Understanding how these enzymes function, within their native lipid environment, is essential for revealing structure–function relationships and informing therapeutic or biotechnological advances.

### 2.3 Interaction of enzymes with biological membranes and materials

**Fig. 2:**
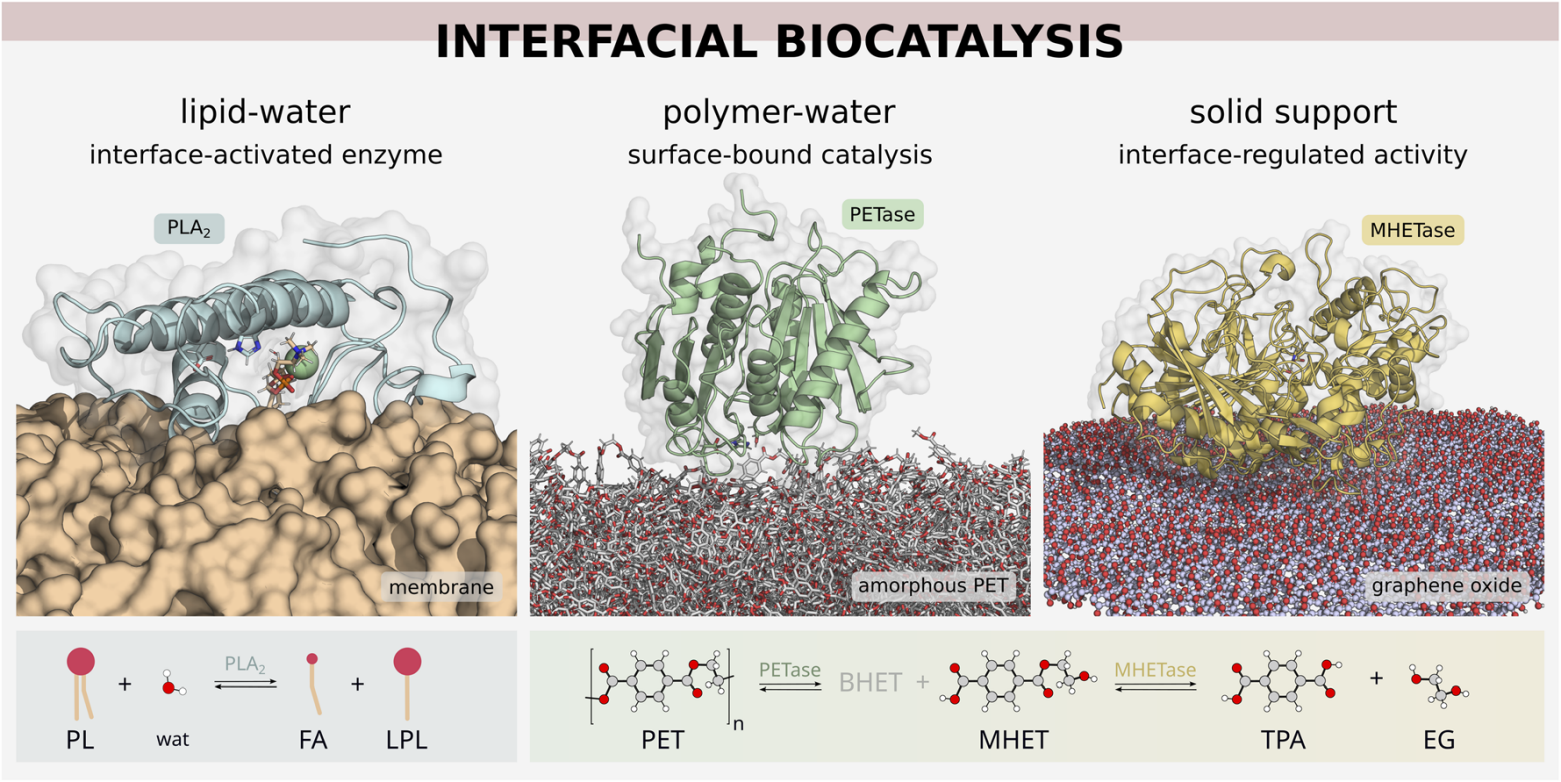
Examples of interfacial biocatalysis, where enzyme activity is governed by interactions with an interface rather than a bulk solution. (i) Hydrolysis of membrane phospholipids by phospholipase A_2_ (PLA_2_) at lipid–water interfaces. PLA_2_ cleaves the ester bond at the sn-2 position of phospholipids (PL), releasing a lysophospholipid (LPL) and a free fatty acid (FA). (ii) Surface-bound hydrolysis of poly(ethylene) terephthalate (PET) by PETase at the polymer–water interface, generating mainly mono(2-hydroxyethyl) terephthalic acid (MHET) and bis(2-hydroxyethyl) terephthalic acid (BHET). (iii) Enzyme immobilization and activity modulation of MHETase at graphene oxide surfaces. In this two-enzyme system, PETase depolymerizes PET to MHET/BHET, and MHETase further hydrolyses MHET to terephthalic acid (TPA) and ethylene glycol (EG).

Many enzyme processes, particularly in industrial contexts, can occur at an interface rather than in bulk aqueous phases (Fig. 2). These include enzymes that are used to capture CO_2_ at the air–liquid interface,^168^ and more recently, significant research effort has focused on enzymes that degrade different plastic polymers, whose reactions occur at the interface between an aqueous phase and a solid phase (Fig. 2).^169–173^

In the context of biological membranes, we also have enzymes that are active at the water–lipid interface (Fig. 2). This is the case, for example, for phospholipase A_2_ (PLA_2_) and phospholipase C (PLC), which hydrolyse lipids in biological membranes.^174,175^ Although these enzymes exhibit limited activity towards non-aggregated substrates in solution, their catalytic activity is triggered upon membrane binding, a phenomenon known as interfacial activation.^176^ For instance, it has been proposed that membrane association may facilitate substrate diffusion along a hydrophobic channel or induce conformational changes in PLA_2_ that facilitate catalysis, or potentially both.^177^

Since these enzymes often exist in a soluble form, their structural characterization has not been as difficult as for transmembrane proteins. However, due to the transient nature of their interaction with membranes, most structural resolution methods often fail to provide information on the most likely interfacial binding site, or on potential conformational transitions upon membrane binding. In this case, computational methods, in particular MD simulations, have proven useful for the characterization of the interfacial binding site of peripheral membrane proteins as well as for modelling the binding mechanism to lipid bilayers.^178^

Several computational studies have addressed the interaction of this class of enzymes with biological membranes. We highlight a recent MD simulation study on the human group IVA cytosolic PLA_2_ (cPLA_2_) that identified key conformational transitions upon membrane interaction, revealing early events that preceded the chemical step of catalysis.^179^ We have recently investigated the catalytic mechanism of both human and snake-venom PLA_2_s, employing full protein-membrane models and using both static and dynamic hybrid QM/MM methods.^177,180^ Conventional MD simulation results showed that membrane composition significantly influences the membrane affinity of these enzymes, highlighting the critical role of lipid composition in modulating the adsorption of peripheral membrane enzymes.^180,181^

Beyond biological membranes, the interaction between enzymes and complex materials introduces challenges that surpass conventional enzyme–ligand modelling approaches. Unlike small molecules, materials such as polymers, nanoparticles, and organic or inorganic surfaces present large and heterogeneous interfaces that can influence substrate accessibility, induce substantial conformational changes in the enzyme, and modulate its stability and dynamics.^182,183^ To accurately capture these events *in silico*, one needs models that account not only for the enzyme's flexibility but also for the structural and chemical properties of the material, which should be thoroughly validated to ensure agreement with its experimentally determined properties. In this context, the choice of force field is a key factor that must be carefully considered. Widely used biomolecular and general-purpose force fields/parameter sets, such as the AMBER force fields (*e.g.*, GAFF, ff14SB, and ff19SB),^184,185^ CHARMM force fields (*e.g.*, C36 and CGenFF),^186^ and OPLS force fields (*e.g.*, OPLS-AA/M),^187,188^ which were developed primarily for small organic molecules and proteins, may not fully capture the intricate nature of nanostructures, extended polymeric surfaces, or heterogeneous interfaces.^189,190^ Still, there are examples where these force fields, either in their canonical form or after reparameterization, managed to accurately reproduce experimentally determined properties, such as glass-transition temperatures and structural architecture, and enabled the study of complex processes including enzyme–surface adsorption.^169,191,192^ In parallel, force fields designed for materials and interfaces, such as ReaxFF^193^ and the INTERFACE force field,^194^ as well as ML force fields,^195^ extend this further, allowing improved modelling of reactivity, complex surfaces, and heterogeneous materials while maintaining computational efficiency.

MD simulations, often complemented by molecular docking and QM/MM calculations, have been instrumental in elucidating enzyme–material interactions at the atomic level. For example, carbon-based materials, including graphene, graphene oxide, and carbon nanotubes, have been widely investigated as supports, providing insights into immobilization, preferred orientations, and structural stability for enzymes such as cytochrome c,^196^ β-lactamase,^197^ α-chymotrypsin,^198,199^ and lipase.^200^ Inorganic surfaces and nanoparticles, including silica and metal oxides, have been studied to understand how surface chemistry and architecture influence binding, conformational dynamics, and, consequently, the catalytic activity of systems such as RNase,^201^ lysozyme,^202,203^ and papain.^204^

Polymers constitute another class of materials that has attracted attention, particularly in the context of plastic biodegradation (Fig. 2). Computational studies on substrates such as polystyrene,^205^ polyethylene,^206,207^ and polyethylene terephthalate (PET)^169,208,209^ have revealed how enzyme adsorption, active site accessibility, and substrate arrangement influence catalytic efficiency. We recently investigated the adsorption process of the PET-degrading enzyme PETase onto large-scale atomistic models of amorphous and crystalline PET using MD simulations.^169^ These calculations supported the experimentally observed preference of PETase for amorphous PET and provided a rationale behind this phenomenon: enhanced surface contacts, improved accessibility of amorphous PET chains to enzymatic attack by PETase, and better organization of the enzyme's catalytic residues when in contact with amorphous PET. The simulations also highlighted key residues and interactions that stabilize the PET chain within the enzyme's active site cavity, offering valuable insight for future enzyme engineering efforts.

Overall, *in silico* studies have clarified how enzyme–material interactions impact catalytic function through advanced and well-characterized enzyme-containing models. The knowledge acquired can also be leveraged to guide the rational design of enzyme–material systems for uses such as biocatalysis and biodegradation.

## 3 Knowledge-based tools to study enzyme mechanisms

### 3.1 Enzyme data and challenges in mechanism annotation

Data about proteins, as available across the literature and public databases, are extensive and rapidly increasing. These data hold great potential for scientific discovery and for the creation of powerful new computational tools. Sequence data, for example, the most abundant type of protein data,^210^ can be used to group proteins into evolutionary and functional families,^211,212^ knowledge that enables the annotation of multibillion-protein metagenomic datasets^213^ and the identification of proteins with unknown function in this so-called biological dark matter.^214^ Structure data are not as comprehensive^215^ but can be equally valuable, as demonstrated by AlphaFold and other deep-learning algorithms (see Section 2.1).^77,87^

Enzyme-specific data, including information on substrate binding,^216^ kinetics,^217,218^ chemical reactions,^219–221^ catalytic residues and mechanisms,^222,223^ complement the sequence and structure to support studies on enzyme function, design and evolution.^224^ One example is reaction data, provided by databases such as Rhea (17,783 reactions),^219^ KEGG (12 324 reactions),^220^ and ExplorEnz,^221^ which maintains the IUBMB (International Union of Biochemistry and Molecular Biology) enzyme list, currently containing 6914 EC (Enzyme Commission) numbers.^225^ While sequence and structure similarities group proteins by ancestry, which is key to understanding divergent evolution, reaction and active site data can additionally reveal convergent evolution, where enzymes with dissimilar sequences and structures independently evolved to catalyse the same reaction through similar or distinct catalytic machinery and mechanisms.^226^

Data on catalytic mechanisms, the focus of this paper, are more complex than other enzyme annotations. A catalytic mechanism is a sequence of chemical steps, each describing one or more bond changes and charge transfers. Unlike the overall reaction, typically defined only by its endpoints (reactants and products), a catalytic step must explicitly specify how these changes occur. For example, a proton transfer between two chemical groups may happen directly or *via* a bridging water molecule. Moreover, to contextualize the mechanism, it is necessary to consider those other annotations, including sequence, 3D structure, substrate and cofactor binding, and the overall reaction, because mechanism description requires mapping active site events onto specific catalytic residues and cofactors, including their position along the sequence and structure.

Another source of complexity is that, for many enzymes, several competing mechanistic proposals exist in the literature, and all must be considered during curation. Finally, unlike other types of enzyme data, such as the PDBx/mmCIF file formats for structure,^227^ or the STRENDA (Standards for Reporting Enzymology Data) guidelines for kinetic data,^228^ there is still no accepted standard for reporting enzyme mechanisms. As a result, many mechanisms reported in the literature are inconsistent, often containing missing steps or atom and charge imbalances. This lack of standardization makes the integration of mechanistic data into databases considerably more difficult, requiring extensive manual curation.

### 3.2 Databases of enzyme mechanisms

The Mechanism and Catalytic Site Atlas (M-CSA)^222^ is a database of catalytic sites and enzyme mechanisms that currently annotates 1003 active sites and the detailed mechanisms of 734 enzymes. Each M-CSA entry has a reference PDB structure and UniProt sequence, as well as a scheme of the overall reaction built from CheEBI (Chemical Entities of Biological Interest) molecules,^229^ and EC-based links to reaction databases. Catalytic residues are identified by their sequence positions in the reference UniProt entry and PDB structure. A textual description of the mechanism and the roles of each catalytic residue is complemented by catalytic annotations using the Enzyme Mechanism Ontology (EMO).^230^

For detailed entries, a full description of the mechanism is given. Catalytic steps are annotated as two-dimensional curly arrow diagrams showing the flow of electrons in the context of the substrate, catalytic residues and cofactors. A text and an EMO annotation of the catalytic role of each amino acid in each step are also given. When multiple mechanistic proposals exist in the literature, they are equally annotated, including which ones have been deprecated, if applicable.

This distinction between “catalytic residue” and “detailed mechanism” entries has an historical origin, since M-CSA was initially created from the merging of the CSA,^230^ a database of catalytic sites, and MACiE,^231^ a database of enzyme mechanisms. However, it also illustrates how knowledge of enzyme mechanisms is typically incomplete, as for many enzymes even if their catalytic residues are known their entire catalytic mechanism is not.

EzCatDB^223^ is another database of enzyme mechanisms that contains annotations on the catalytic mechanisms of 877 enzymes. Mechanisms in EzCatDB are grouped into a hierarchical classification of catalytic mechanisms called RLCP. This is a four-level classification reminiscent of the EC classification where each character describes one of the four levels of the classification: Reaction, Ligand group involved in catalysis, Catalysis type, and residues/cofactors located on Proteins.

### 3.3 Leveraging existing mechanistic knowledge to study new enzymes

Protein similarity, in terms of conserved sequence and structure, provides evidence for shared ancestry and is suggestive of conserved function.^224^ This is particularly true for orthologous proteins, which arise from gene duplication during speciation events and remain subject to similar evolutionary pressure in both species. These relationships are especially marked in the active sites of enzymes, which are extremely well conserved throughout evolution. For metabolic enzymes, whose pathways tend to be shared across most living organisms, it is common for enzyme function to be conserved across all domains of life,^232^ which implies that the conservation and functional history of some of these enzymes can be traced back to LUCA (Last Universal Common Ancestor), the ancestral population of single-celled organisms from which all life on Earth descends. In general, when it comes to the catalytic mechanism, it is reasonable to assume that enzymes sharing the same active site and catalysing the same reaction also follow the same catalytic mechanism, although this hypothesis has not yet been systematically tested.

From this evolutionary perspective, the most straightforward and logical starting point for studying the mechanism of a given enzyme is examining proteins with similar sequences and conserved catalytic residues, particularly when they catalyse the same overall reaction. The databases discussed above are essential tools for establishing these relationships. In fact, the extrapolation of mechanistic annotations to enzymes with identical active sites and reactions, when applied to the M-CSA dataset, expands the 734 manually curated mechanisms to more than 73 000 enzymes in Swiss-Prot.

Beyond these clear-cut cases, knowledge-based approaches are also proving useful in revealing potential mechanistic hypotheses for dissimilar enzymes. The active sites of enzymes typically consist of a small number of amino acids and cofactors, and only a limited subset of the twenty proteinogenic amino acids participate directly in catalysis.^233^ As a result, redundancy in the types of chemical transformations catalysed by enzymes is expected. Enzymes may therefore share mechanistic components, even when they are not evolutionarily related or even if they catalyse different overall reactions. By identifying these recurring mechanistic features, or rules of catalysis, it is possible to generate mechanistic hypotheses for any active site, provided that it shares at least some active site similarity with the known catalytic systems.

We are aware of three recently developed computational tools, each created independently, that follow this same general approach to predicting the mechanisms of uncharacterized enzymes: EzMechanism,^234^ MechSearch,^235,236^ and MechFind.^237^ The appearance of these three tools in rapid succession highlights the growing interest in automating the difficult task of studying enzyme mechanisms and was made possible by the availability of mechanistic data in machine-readable form in the most recent version of M-CSA. More generally, this illustrates the importance of the FAIR (Findable, Accessible, Interoperable, and Reusable) principles, since it was the standardization and ready availability of these data, previously scattered throughout the literature, that made new approaches such as these possible.

**Fig. 3:**
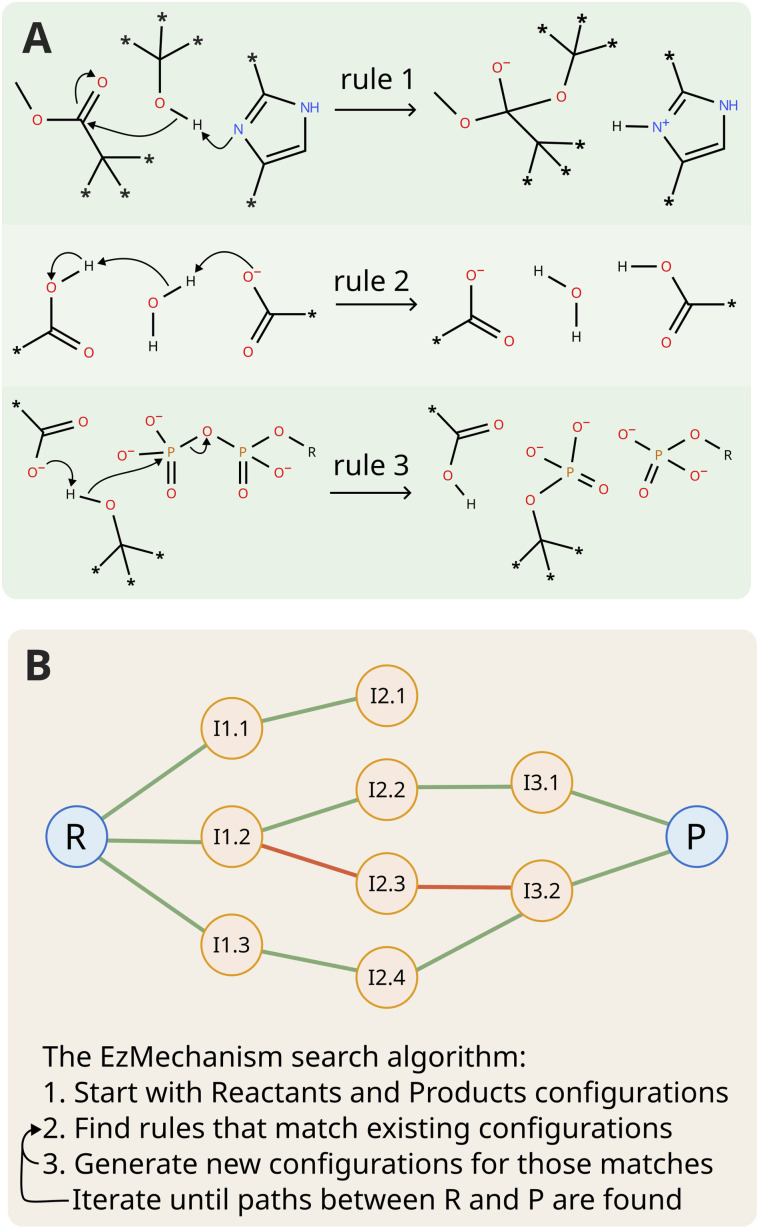
(A) Examples of catalytic rules extracted from the M-CSA database. Asterisks indicate positions that can match carbon or hydrogen atoms. (B) An illustrative example of a mechanistic graph showing active site configurations as nodes and catalytic steps as edges. The reactant and product configurations are represented in blue and reaction intermediates in orange. Green edges indicate fast catalytic steps, with a low energy barrier, while red edges represent steps with inaccessible catalytic barriers.

In the case of EzMechanism,^234^ a set of 7218 catalytic rules is used to generate mechanistic proposals based on the 3D structure of the active site and the overall reaction catalysed by the enzyme.^234^ These rules, exemplified in Fig. 3A, capture the bond changes that can occur when a set of chemical groups is found in the active site. Rule 2 in the figure, for example, states that if a protonated carboxylic group (as found in Asp, Glu or any substrate or cofactor; the rules are not specific to a particular molecule) is found close to a non-protonated carboxylic group and a water molecule, then the proton may be transferred from one group to the other through the water molecule. Even simple rules like this, when combined, can generate pathways that lead from the reactants to the products, as seen in Fig. 3B. In this graph, nodes are chemical configurations of the active site corresponding to the reactants, products and intermediate states, while edges are catalytic steps that transform one configuration into another. A catalytic mechanism is defined as any path of catalytic steps that link the reactants to the products (paths R- > I1.2- > I2.2- > I3.1- > P and R- > I1.3- > I2.4- > I3.2- > P in Fig. 3B). The depicted graph shows other interesting features seen in this kind of mechanistic network, such as dead-ends (R- > I1.1->2.1), inaccessible configurations and paths (I1.2- > I2.3- > I3.2), and bifurcations (I1.2 and I3.2).

EzMechanism was tested on 56 enzymes from M-CSA and six additional enzymes not included in the reference dataset. When using the catalytic rules extracted from the mechanism being tested, even if mixed among all the other rules, the method can recover the correct mechanism for virtually all tested enzymes. However, for roughly half of the enzymes, at least one catalytic rule is unique to that enzyme. This means that when applied to uncharacterised enzymes, EzMechanism may not be able to recover the full pathway for about half of the enzymes, unless a close homologue is already represented in the dataset. A practical way forward involves generalizing certain rules by reducing their specificity. This can be done by defining a covalent environment around reaction centres that includes fewer atoms.

MechSearch,^235,236^ a method that predates EzMechanism, uses a formal graph-transformation framework to propose mechanisms for a given reaction and set of catalytic residues. Although implemented independently, MechSearch shares many of the concepts introduced above, including a similar implementation of catalytic rules and the ability to generate new rules by composition. MechSearch does not require 3D structural information of the active site, taking only a list of catalytic residues and the reaction as input. It has been tested on RHEA reactions and successfully generated plausible mechanisms for several of them using different combinations of catalytic amino acids.

MechFind,^237^ a more recent tool, encodes catalytic steps as vectors of gains and losses of chemical moieties, a conceptual approach distinct from the tools discussed above. MechFind abstracts the mechanism-prediction problem even further than MechSearch, as it does not require 3D coordinates of the active site or even its amino acid composition. From the overall chemical reaction alone, MechFind can propose candidate mechanisms and infer which catalytic residues may be required, an attractive feature for *de novo* enzyme design. The software was tested on approximately 15 000 RHEA reactions and was able to propose mechanisms for 57% of them. Both MechSearch and MechFind are standalone programs, with source code available in GitHub, while EzMechanism works as a server, integrated in the M-CSA website.

An interesting and common outcome across EzMechanism, MechSearch, and MechFind is that they typically produce not one but multiple alternative catalytic pathways for a given reaction. This raises the problem of identifying which path is correct. Some heuristics, like minimizing the number of catalytic steps or prioritizing shorter interatomic distances for newly formed bonds, can help rank alternatives but, ultimately, discriminating among competing proposals requires QM/MM calculations, as discussed in this review. A new version of the EzMechanism software is currently being developed to streamline the setup, execution, and analysis of QM/MM calculations so that many alternative pathways suggested by the algorithm can be tested efficiently.

It has also been argued recently^238^ that at least some of these alternative proposals may not be “wrong,” but instead represent viable routes that enzymes can follow, depending on stochastic fluctuations in the active site or larger-scale conformational changes that favour one path over another. Typical QM/MM studies do not exhaustively explore this broad mechanistic space because of computational cost and the burden of preparing and analysing many calculations, although this may become feasible with our upcoming update.

## 4 Characterizing reaction mechanisms with hybrid QM/MM methods

### 4.1 Computational methods in the study of reaction mechanisms

Computational methods have emerged as indispensable tools for the atomistic characterization of enzymatic reaction mechanisms, providing insights often unattainable with traditional experimental techniques. *In silico* experiments allow the testing of alternate reaction pathways that may not be evident from available experimental data as well as a detailed description of the transient events that occur during a chemical reaction, including energy profiles of each mechanistic step, structures of TSs, intermediates with short half-lives and their associated electronic properties, and minimum energy paths describing the most favourable reaction coordinates for catalysis. This information is invaluable for understanding how enzymes accelerate complex chemical reactions and how they stabilize or destabilize stationary points. The calculated free energy barriers can subsequently be compared with kinetic data through transition state theory (TST). In its conventional form, TST relates the rate constant to the free-energy barrier of the rate-limiting step according to the Eyring equation, assuming no recrossing of the transition state occurs. In variational formulations (VTST), the equation expresses the catalytic rate as the product of a transmission coefficient, *k* (which accounts for deviations from conventional TST behaviour, including nonequilibrium effects, recrossing of the transition state or quantum tunnelling), and a TST rate constant generally expressed in terms of the free-energy barrier Δ*G*^‡^ of the rate-limiting step.^239,240^ It is also useful, for instance, for follow-up drug discovery^241,242^ and enzyme engineering campaigns.^243^

As mentioned earlier, electronic structure-based methods are commonly used to study bond-breaking and bond-forming events and to calculate the energetic cost associated with these phenomena. For the study of enzyme catalysis, given the size of the computational model often needed for such reactions, DFT and SE methods are the most viable options to overcome the enormous computational cost of the most accurate wave function theory (WFT) methods. The trade-off between accuracy and computational feasibility is a challenge computational (bio)chemists must constantly navigate. Nonetheless, when appropriate care is taken in choosing an appropriate DFT functional/basis set combination or SE method, good and meaningful results can be achievable.^244^ WFT methods are commonly used for single-point energy corrections on geometries previously obtained with DFT or SE approaches. In particular, the domain-based local pair natural orbital coupled cluster with single, double, and perturbative triple excitations (DLPNO-CCSD(T)) and the Spin-Component-Scaled Second-Order Møller–Plesset Perturbation Theory (SCS-MP2) are now routinely used to refine reaction thermodynamics.^245–247^

The practice of blindly choosing a functional based on popularity may lead to significant errors in some systems.^248^ For this reason, consulting available benchmarks for the reaction of interest is imperative, and if they are not available, assessing more accurate *ab initio* WFT or hybrid WFT/DFT methods on a minimal computational model and comparing the values to those obtained from a selection of DFT or SE methods are recommended.^249^ This is particularly important when the catalytic mechanism involves complex, multi-step chemical reactions, such as redox reactions with or without cofactors and metal centres, or radical species. In these complex cases, different DFT or SE methods may return energy profiles that differ significantly.^245,250^ In any case, researchers should study the catalytic site and possible mechanisms and assess which charge/multiplicity combinations may be visited throughout the reaction and evaluate them accordingly. This is particularly relevant for systems involving metals that undergo different oxidation states with multiple electronic configurations during the reaction and for radical species.^251–257^ For metal-containing systems, in particular those involving transition metals and heavy elements, relativistic effects may play an important role and should also be accounted for.^258^

Despite being around for decades, both DFT and SE methods have seen recent developments, demonstrating that the field remains active and innovative. Most notably, the continuous development and improvement of dispersion correction methods aims to tackle one of DFT's major limitations, the lack of long-range electron correlation effects responsible for London dispersion forces. The recently developed DFT-D4 model represents a significant advancement over its predecessor,^259^ while novel approaches such as density and dispersion-corrected DFT address density delocalization errors and dispersion errors simultaneously.^260^ Likewise, SE methods have been continuously improved with the goal of reaching DFT-level accuracy while maintaining substantially smaller computational costs. The GFN-xTB family of methods, particularly GFN2-xTB, exemplifies this progress by incorporating new features such as anisotropic multipolar contributions and self-consistent dispersion corrections with the D4 model, achieving significant improvements while remaining faster than conventional DFT calculations.^261,262^

Even though DFT and SE methods are computationally efficient, and computational power has been steadily increasing as increasingly efficient HPC clusters are developed, they are still extremely expensive for describing an entire enzyme, although this has been achieved for the calculation of binding energies.^263^ DFT simulations of thousands of atoms were previously unfeasible and only very recently became possible, thanks to the combination of more linearly scalable code with graphics processing units (GPUs).^264–266^ Those large models are still not routinely used; typically, an enzyme model treated with DFT potentials remains restricted to no more than 300 atoms, and SE methods can handle systems of up to 1000 atoms, with larger systems possible with current GPU linear-scaling codes. The computational cost increases with the complexity and number of steps of reaction and the number of alternative mechanisms being investigated.

**Fig. 4:**
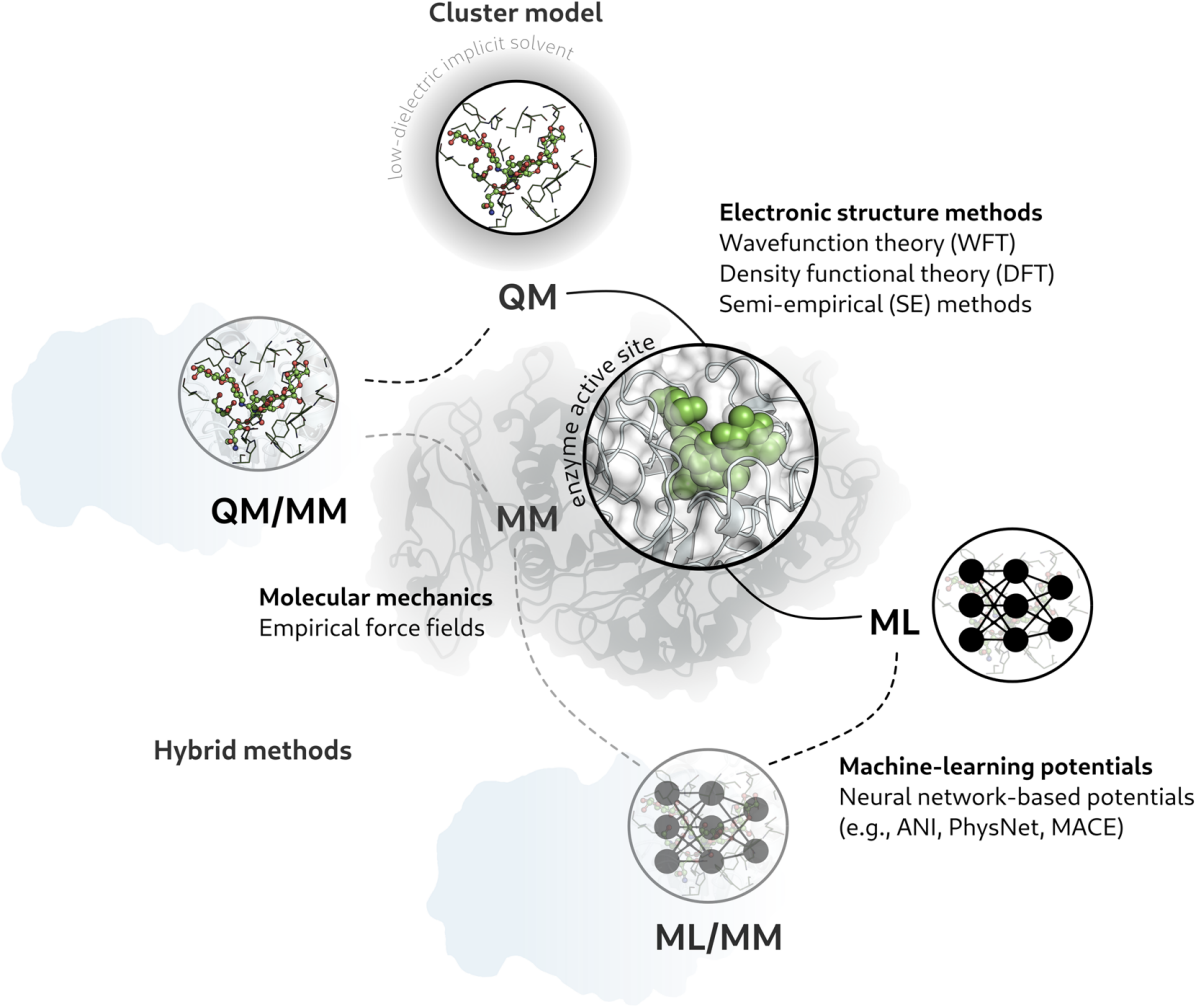
Schematic representation of computational methods used to study enzyme reaction mechanisms. Enzyme reaction mechanisms are commonly investigated using electronic structure methods, either through QM cluster models or hybrid schemes such as QM/MM, in which the enzyme environment is treated with classical force fields. More recently, ML potentials have emerged as an efficient alternative to QM methods, leading to ML/MM hybrid approaches, although application to enzyme systems remains limited.

There are two main widely used solutions that researchers adopt to tackle this problem and simulate reactive enzyme systems (Fig. 4): the cluster model approach^267^ and the QM/MM hybrid approach.^268^ In the cluster model approach, only a fraction of the whole enzyme is explicitly considered. The constructed model should contain the active site and any residues that participate in important interactions for the reaction to occur, while the rest of the enzyme is mimicked by a low-dielectric implicit solvent (*e.g.*, dielectric continuum models). The residues at the edges of the model, which have bonds cut, are typically capped, most common with hydrogen atoms, and kept restrained to maintain a coherent conformation.^269^ This method has seen great success in the past decade and has been used to elucidate catalytic mechanisms of multiple enzymes, including complex cases involving radicals and metal centres.^270,271^

**Scheme 1:**
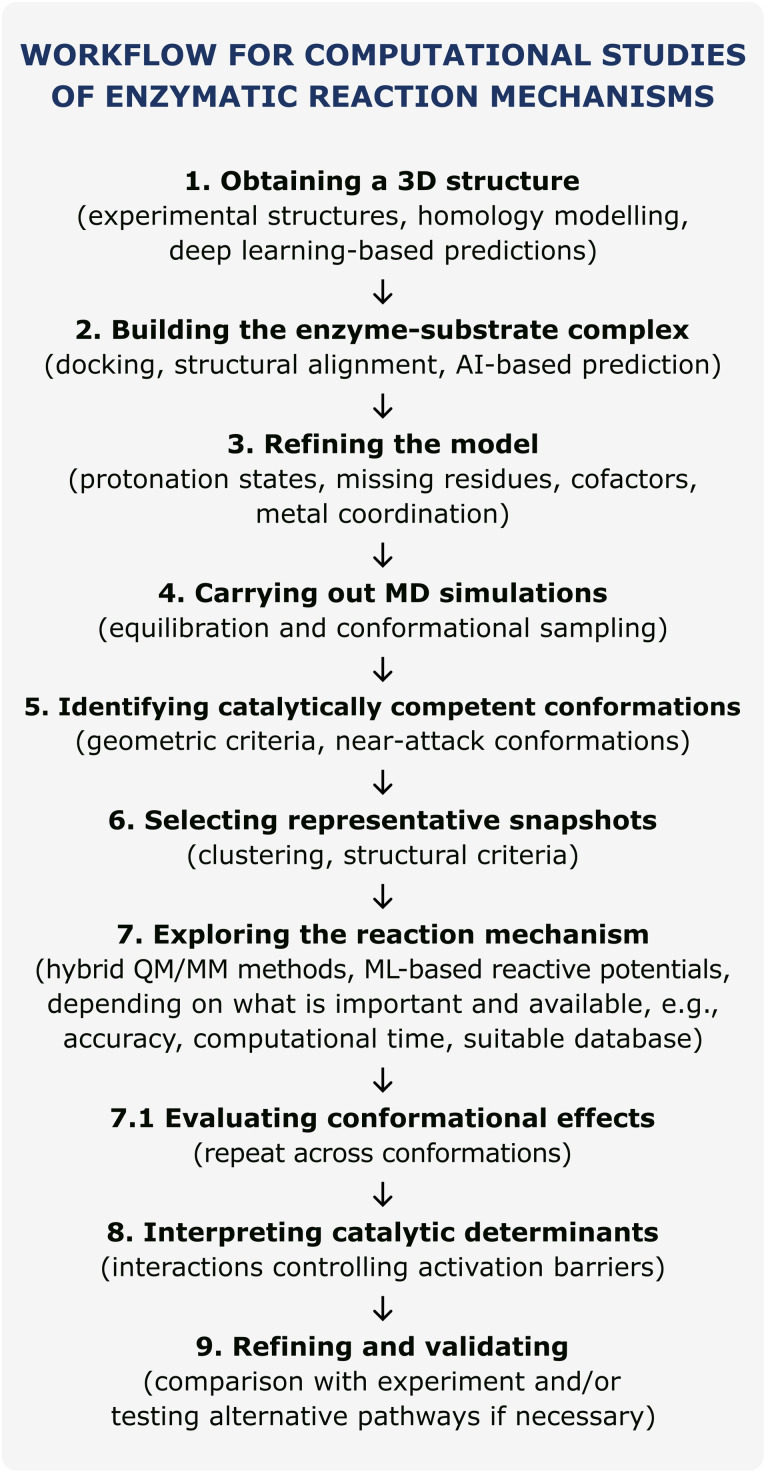
General workflow for computational studies of enzymatic reaction mechanisms. The process typically begins with obtaining a 3D structure, followed by model preparation and conformational sampling through MD simulations. Representative conformations are then used to explore the chemical step using QM/MM hybrid methodologies or ML-based reactive potentials. Iterative refinement and comparison with experimental data are sometimes required to distinguish between alternative mechanistic hypotheses.

Given the relevance of the protein environment to the reaction, the entire enzyme can be explicitly simulated using the hybrid QM/MM method, which has become the most popular approach for studying enzyme reactivity. In this approach, the reactive region of the enzyme is treated with a DFT or SE method analogous to the cluster model, while the remaining enzyme residues and solvent are described by classical MM. Using this formalism, the total energy of the system is obtained through one of two main schemes: (1) the subtractive scheme, most notably ONIOM, which sums the MM energy of the whole system and the QM energy of the reactive region and then subtracts the MM energy of the reactive region;^272^ (2) the additive scheme, which sums the MM energy of the non-reactive region with the QM energy of the reactive region and adds a term accounting for van der Waals, electrostatic, and bonded interactions across the two regions.^273^

The setup of the QM/MM model is of utmost importance. This approach involves critical aspects that may be significant sources of error and therefore need to be carefully addressed. Defining the reactive region to be treated with a QM Hamiltonian necessarily involves cutting covalent bonds at the QM/MM boundary. In addition to selecting all atoms directly participating in the reaction and those influencing it through important interactions, boundaries should avoid cutting polarized bonds or rings; single carbon–carbon bonds are preferred to minimize errors, and the boundary should not disrupt crucial electrostatic interactions.^274^ The valence of the dangling QM atom is usually capped with a hydrogen atom, using the link-atom approach, although more sophisticated solutions have also been developed, such as frozen orbitals^275^ and pseudobond^276^ methods. The most common scheme in non-polarizable force fields for treating electrostatic interactions across the QM/MM boundary is electrostatic embedding, in which the surrounding MM environment is incorporated into the QM Hamiltonian as point charges, enabling the polarization of the QM subsystem by the MM environment while MM charges remain fixed.^277^ This offers superior accuracy over mechanical embedding, where electrostatics are accounted for only at the MM layer and the QM electrons do not respond to their environment. Nonetheless, if there is a high charge density near the boundary (*e.g.*, a cut salt bridge), significant errors may arise. More recent polarizable embedding schemes enable mutual polarization between QM and MM regions,^278,279^ providing the most accurate representation but at the expense of requiring properly parameterized polarizable force fields and higher computational requirements.^280^

Enzymatic QM/MM models are often derived from structures equilibrated under periodic boundary conditions, and subsequent calculations may either retain a periodic explicit environment using the QM/MM–Ewald approach, or use a spherical cut of the system having a variable radius centred on the active site with a finite solvent shell, the droplet model. These choices affect the treatment of long-range electrostatics and the extent to which the protein environment is allowed to relax. Truncated models require careful handling of MM charge cutoffs and restraints to avoid unphysical relaxation of the truncated system. Since enzymatic reaction energies can be sensitive to electrostatic preorganization, these conditions should be, whenever possible, assessed through convergence tests. In principle, if handled with care, the energy differences obtained using each approach should be very small.^281^

The recent successes of machine learning interatomic potentials (MLIPs) learned from QM data in the field of homogeneous and heterogeneous catalysis^282–286^ have highlighted the potential of these representations for the accurate description of molecular properties with accuracies at times comparable to those of the most accurate electronic structure methods developed to date. As we push their limits towards the representation of larger systems, as is the case for enzyme systems, hybrid ML/MM (Fig. 4) schemes are also becoming more popular.^287,288^ We will address these topics in more detail in the subsequent section.

### 4.2 Modelling of enzyme reaction mechanisms

#### 4.2.1 Static representation of a reactive enzyme

The modelling of an enzyme reaction with QM/MM may be performed using static QM/MM or may be combined with conformational sampling methods, most notably MD. Adiabatic mapping of enzyme mechanisms typically begins with modelling a catalytically competent enzyme–substrate complex, often surrounded by a shell of water molecules. The initial starting point is typically selected after a cluster analysis of MD trajectories that considers critical catalytic distances, or by directly using the X-ray structure when quasi-catalytic states are crystallized. The direct use of X-ray structures should be done with care, as these often do not represent the catalytic geometry of the complex.^289^ To simplify the calculations, atoms distant from the catalytic site are frequently frozen to reduce the number of degrees of freedom.^290^ Then, tentative reaction coordinates corresponding to the atomic motions involved in each mechanistic step are defined to yield an initial potential energy profile for the reaction, from which tentative stationary points can be identified: minima can be optimized to locate reactants, intermediates and products, whereas maxima can be used as tentative TS guesses to pursue optimization. Once TSs are optimized, a vibrational frequency analysis can be carried out to identify the single imaginary frequency corresponding to the reactive mode, from which the associated minima can typically be found by intrinsic reaction coordinate calculations.

The representation of an enzyme as a single-conformation QM/MM model has been a popular approach and is still widely used, with good results.^247,251,254,291–294^ This approach heavily relies on the careful choice of the starting structure and assumes that, if a representative structure of the reactive enzyme–substrate conformation is used, often referred to as a near-attack conformer (NAC), the obtained energy profile can represent a possible catalytic pathway followed by the enzyme.^254^ This is justified because the enzyme-catalysed reaction occurs from conformations close to the NAC,^295^ which are sporadic.^296^ It also assumes that chemical transformations, not conformational sampling, are rate-limiting, making the adiabatic energy profile representative of catalysis.^297^ Single conformation QM/MM calculations are nevertheless susceptible to bias, since the reaction barrier calculated from one geometry may not reflect the catalytic behaviour of the enzyme. This strongly depends on whether the selected conformation is catalytically competent or not. The catalytic efficiency of enzymes depends on maintaining catalytically competent geometries in favourable conformations, which is enabled by their inherent structural flexibility. Although appropriate distances and orientations within the active site–substrate complex are necessary, they are not sufficient, as medium- and long-range organisation of the enzyme can also contribute significantly to the catalytic barrier and is harder to predict. This conformational heterogeneity can exert an impact on the computed catalytic barriers and remains a challenge to capture computationally.^298–301^ Consequently, single-conformation approaches neglect enthalpic and entropic contributions from conformational heterogeneity.

The influence of dynamical fluctuations on enzyme catalytic activity remains a matter of debate. Single-molecule studies have shown significant fluctuations in enzyme barriers, demonstrating that the catalytic rate, traditionally considered as a fixed value, is, in fact, an average over an ensemble of conformationally dependent rate constants. These results underscore the importance of studying different enzyme conformations during enzyme catalysis modelling.^300,302,303^

So far, no universal computational approach exists that captures all dynamic effects of an enzyme within a biologically relevant timescale.^304^ To mitigate these limitations, the multi-conformation QM/MM (multi-PES) approach extends the use of adiabatic mapping to an ensemble of geometries. In a multi-PES approach, the workflow begins with the exploration of the enzyme–substrate complex conformational space, which is usually achieved *via* classical MD simulations, over timescales ideally close to microseconds as to span meaningful enzyme–substrate rearrangements. Enhanced sampling methods, such as metadynamics,^305^ replica-exchange MD,^306^ or accelerated MD,^307^ can also be employed to more efficiently capture rare conformations that classical MD might miss. These methods can produce a large ensemble of structures with variations from global conformational changes to small local rearrangements, which would normally be inaccessible to QM/MM MD strategies due to computational cost. Representative snapshots are extracted from these simulations and treated as a distinct microstate for which an independent QM/MM potential energy profile is calculated within a static framework. This method generates multiple PESs that convert structural heterogeneity into a distribution of activation energies.^308–315^

Although temporal fluctuations within individual snapshots are not included, analysing an ensemble of conformations provides an improved representation of the population of accessible states. This approach helps to distinguish catalytically competent geometries from non-competent ones and provides mechanistic insights beyond those of single-conformation QM/MM approaches. It also clarifies how structural variations, such as active site geometry or hydrogen bonding networks, influence the reaction barrier.^316^ The final energy barrier can be computed by averaging the barriers from multiple conformations, according to different averaging schemes reported in the literature:^317^ the arithmetic average, where all conformations contribute equally;^308,313,318–320^ the Boltzmann-weighted average, where lower-energy conformations dominate;^310,321^ the minimum-energy approach, which assumes that only the lowest barrier is catalytically relevant;^322^ and the exponential average, which is mathematically more consistent with TST.^323–326^

A common challenge with the multi-PES QM/MM approach is defining criteria for selecting initial snapshots from the MD simulations, since the number of conformations in a trajectory is too large to be studied exhaustively. Selecting a conformation based only on its total energy is not a reliable strategy, since the activation energy is not a state function of the reactant energy, and the energy of the reactants is therefore not directly correlated with the activation energy in enzymatic reactions. The most common approach relies on clustering structures according to structural motifs or geometric features that are important for catalysis, or alternatively selecting snapshots equally spaced in time or randomly. An immediate difficulty that arises is establishing the minimum number of conformations required to achieve a reliable averaged barrier. According to a study by Ryde,^327^ exponential averaging does not converge reliably, as a limited subset of low-energy conformations can disproportionately influence the calculated average. The variance of the energy distribution serves as a useful metric for estimating the reliability and expected accuracy of the resulting value, as well as for estimating the required number of energy barriers for a given accuracy. However, practical experience in QM/MM studies often suggests that barrier distributions are not symmetric but skewed toward higher energies, with catalytically competent conformations yielding barriers close to the experimental value, while suboptimal conformations tend to produce larger barriers. In such cases, the variance alone does not fully describe the distribution, and sampling on the order of ∼10 starting conformations is often sufficient to obtain a stable estimate of the barrier. In conclusion, in practice, the way the energy barriers are averaged, together with the criteria used to select the snapshots and the number of conformations considered, can strongly influence the results.

The multi-PES QM/MM approach has been applied to study key enzymes in the HIV-1 life cycle, namely HIV-1 integrase and HIV-1 protease. For HIV-1 integrase, calculations based on five MD-derived snapshots produced activation energies ranging from 7.6 to 17.4 kcal mol^−1^, spanning a range of nearly 10 kcal mol^−1^.^309^ This approach aimed to test whether the most likely mechanistic proposal, the hydroxide-base mechanism, remained consistent, regardless of the starting enzyme–substrate geometry. This was motivated by single-molecule experiments^304,328^ and the concept of dynamic disorder, where conformational fluctuations within a given folding state, occurring on timescales comparable to catalytic turnover, influence reaction rates (*k*_cat_). Although sampling was limited, it was sufficient to demonstrate how conformational diversity can modulate reactivity and underscore the need for careful consideration of this effect in static QM/MM studies of enzymatic reactions.^309^

Similarly, studies on HIV-1 protease using the multi-PES approach revealed how the conformational space of this enzyme influences its reaction rate and mechanism. In a seminal study by Carloni and coworkers, large-scale protein fluctuations involving the flaps and cantilever of the HIV-1 protease structure were shown to affect the conformations of the substrate at the cleavage site and in turn modulate the activation free energy barrier of the enzymatic reaction.^329^ More recent work examined how small-scale fluctuations at the active site, and not large-scale protein motions, could influence reaction rates. By analysing the first step of the catalytic mechanism of HIV-1 protease across 40 conformations equally spaced in time, from replicate MD simulations at the nanosecond timescale, activation free energies ranging from 14.5 to 41.1 kcal mol^−1^ were obtained, yielding an apparent barrier of 16.5 kcal mol^−1^, very close to the experimental value of 15.9 kcal mol^−1^. Distinct configurations adopted by the active site and different reaction mechanisms were also observed, and simple geometric descriptors shown to correlate well with differences in the corresponding activation barriers. In addition, the electrostatic environment provided by the enzyme was found to impact transition stabilization within just a few nanoseconds.^314,326^ Extending the same multi-PES approach to an enlarged QM region enabled the identification of two distinct reaction mechanisms with similar reactant probabilities and barrier heights, both leading to the same gem-diol intermediate, with activation energies spanning 17.3–32.2 kcal mol^−1^. Catalytically competent and incompetent conformations were also discriminated, and local hydrogen bond rearrangements in the active site were shown to determine which reaction the enzyme follows.^314^ This mechanistic divergence was later confirmed using QM/MM MD simulations.^330^ An important advantage of the multi-PES approach is that it can help identify the interactions responsible for catalysis and for the preferred reaction mechanism. By comparing conformations that produce low and high activation barriers, it becomes possible to pinpoint structural and electrostatic features that favour TS stabilization and thus obtain chemically meaningful insight into catalytic preorganisation.

In addition to enzymes relevant in the HIV-1 life cycle, the multi-PES QM/MM method has been applied to many other enzymes across different classes.^294,315,317,331–335^ These studies demonstrated how small rearrangements in the active site can impact reaction rates *via* the different reaction barriers calculated for multiple structures. They thus help identify geometric descriptors relevant to these diverse enzymes and highlight how destabilization of optimal descriptors can influence reaction barriers.

#### 4.2.2 Molecular dynamics study of a reactive enzyme

The adiabatic mapping approaches just discussed rely heavily on the choice of the initial structure, although this can be partially mitigated by using multiple starting conformations extracted from MD trajectories.^315^ These approaches, however, do not properly account for thermal sampling and often underestimate dynamic contributions to chemical steps.^330,336^ MD simulations are therefore needed to overcome the lack of thermal sampling while also providing a more realistic representation of the thermodynamic conditions under which reactions take place. Besides missing important conformational changes, static QM/MM cannot provide true free energies but rather relies on approximate treatments using the particle-in-a-box, rigid rotor, and harmonic oscillator formalisms to estimate electronic, rotational, translational, and vibrational contributions to the Gibbs energy. These equations assume the whole model behaves as an isolated non-interacting particle, which introduces systematic errors, especially for translational and rotational entropies.^337^ The limitations of these approximations can become more evident when considering a single conformation for reactive enzyme systems and given the fact that such complex systems often exhibit many low vibrational modes, which can introduce errors typically within 2–3 kcal mol^−1^. Nonetheless, these limitations can be overcome by expanding the number of reactive conformations under consideration, carefully treating low-frequency modes, and ensuring that characterization of stationary states includes validation through vibrational analysis.^327,338^

Born–Oppenheimer and Car–Parrinello MD have been developed and successfully implemented over the past few decades to describe molecular systems within the adiabatic approximation, effectively capturing the coupling between nuclear motion and the ground-state electronic structure.^268^ These methods have enabled the study of a wide range of chemical processes, from acid–base reactions, group transfers, and nucleophilic substitutions to processes that involve changes in the electronic structure.^339–343^ Nevertheless, these approaches remain unable to simulate the timescale of conformational changes coupled to chemical events at a QM level since the electronic detail required to describe bond formation and breaking events rapidly increases the complexity of the molecular Hamiltonian, severely hampering the efficiency of current simulation codes. SE methods can be used in combination with hybrid QM/MM approaches to speed up the calculation of the electronic Hamiltonian and reduce the computational cost, as the search for more scalable implementations providing accurate descriptions of the electronic structure continues. These methods are not universally transferable, as they may fail to consistently describe different chemistries, but they simplify the electronic structure calculation, making simulations over longer timescales feasible.^42^ Popular methods, such as AM1, PM6 or the conceptually different SCC-DFTB, have been used to study several mechanisms involving acid–base, nucleophilic substitution, or group transfer reactions with a good degree of success.^344–347^

The empirical valence bond (EVB) method, developed by Warshel and coworkers,^348,349^ is another alternative to address the sampling problem in the study of enzyme reaction mechanisms. The EVB method builds the potential of a system as a linear combination of relevant diabatic states (reactants, products, and intermediates), where these states are described at a classical level and their couplings are described with carefully parameterized terms. With this, the free energy of adiabatic transformations can be obtained by interpolating along the effective potential energy function connecting the reactant and product states.^350^ Since the quality of the method output is heavily dependent on the parameterization of the EVB potential, high-quality experimental or accurate *ab initio* data are required to calibrate both the diabatic state parameters and their coupling elements, making this step extremely important.^351^ EVB has been extensively applied to study reaction mechanisms both in solution and in enzymes,^352^ thereby emphasizing the dominant role of electrostatic preorganization, rather than conformational dynamics, in lowering activation barriers relative to the corresponding reactions in solution.^301,353^ In addition, it has also been used to calculate enthalpies and entropies of enzyme-catalysed reactions^354–356^ and to address their temperature dependence,^357,358^ or to screen engineered mutants.^359,360^

### 4.3 Enhanced sampling applied to the study of reaction mechanisms: improving the exploration of the reaction conformational space

Current conventional QM-based MD simulations typically reach only a few tens of picoseconds at the DFT level and a few nanoseconds at the SE QM level, with the achievable timescales strongly dependent on the system size, available computational resources, and the chosen level of theory. This is a severe limitation when studying enzymatic reactions, which often involve crossing substantial free-energy barriers (typically in the range of 10–25 kcal mol^−1^),^361^ making transitions between metastable states rare within the timescales accessible in QM/MM MD simulations.

This sampling bottleneck can be understood quantitatively through the lens of statistical mechanics: in the canonical ensemble, the probability of a given configuration **R** is distributed according to the Boltzmann distribution:1
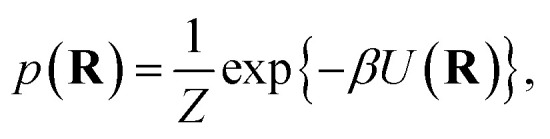
where *β* = 1/*k*_B_*T*, with *k*_B_ being the Boltzmann constant and *T* the temperature, *U*(**R**) is the potential energy of the system, and *Z* is the corresponding partition function of the system. This distribution is typically multimodal, with high-probability regions corresponding to free-energy minima associated with metastable states (*e.g.*, reactants, products, or intermediates), separated by low-probability regions corresponding to large free-energy barriers associated with TSs. At the timescales accessible in QM/MM MD, a simulation initiated in one such metastable basin tends to remain trapped there for the entire duration of the calculation. Even if a barrier crossing occurs, it is rarely observed more than once, leading to poor sampling. Thus, no thermodynamic or kinetic quantities can be computed with statistical confidence, and the reaction mechanism can only be partially explored.

To accelerate the sampling of rare events, several enhanced sampling techniques have been proposed to allow the observation of reactive processes within affordable computational times.^362^ More importantly, for the study of reaction mechanisms and reactive transformations in general, enhanced sampling can provide access to the underlying thermodynamics and, in some cases, kinetics of the reactive process, providing insight into the molecular determinants of reactivity.^363,364^

**Fig. 5:**
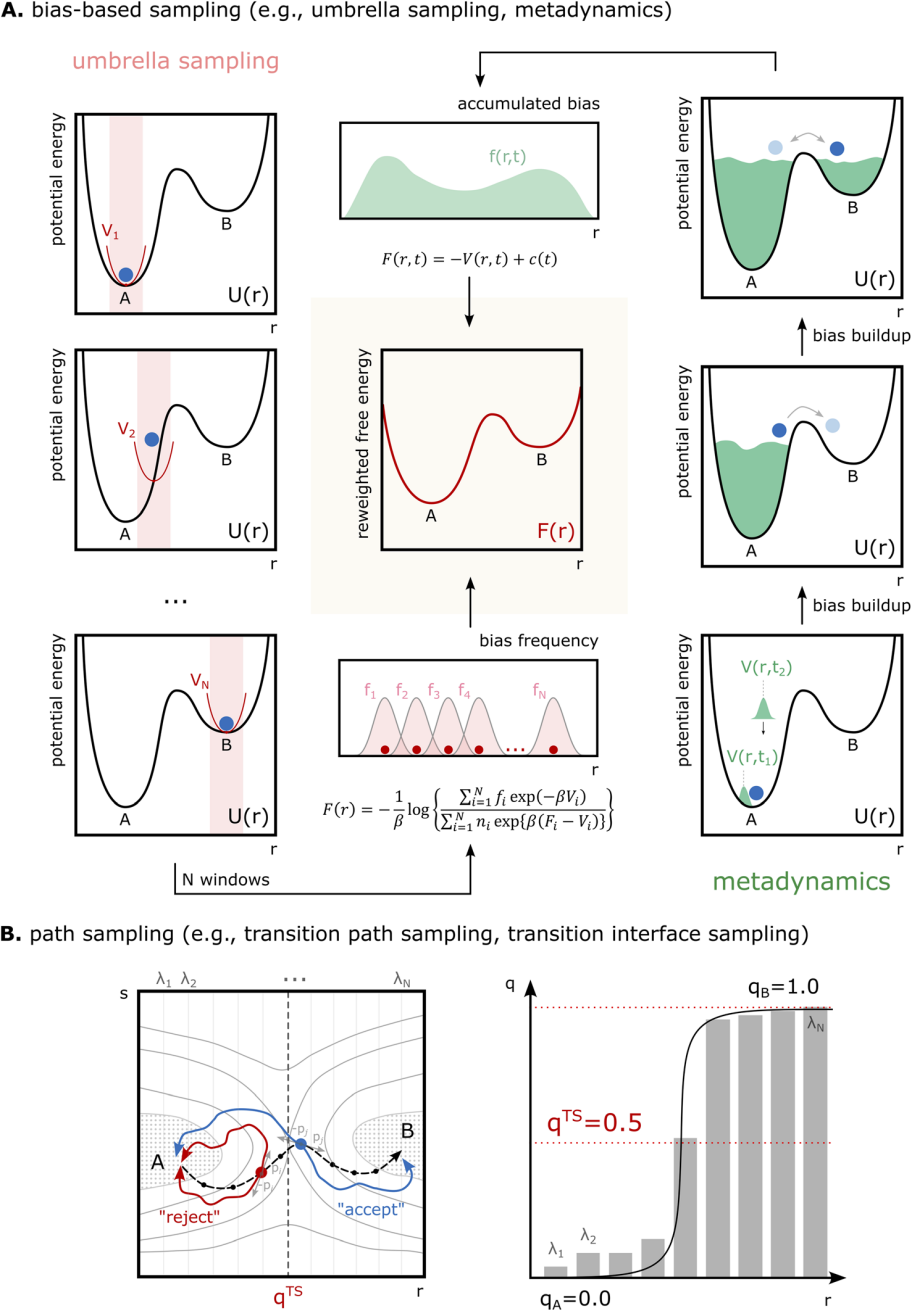
Schematic representation of enhanced sampling techniques involving: (A) biasing of the PES to promote rare transitions; (B) path sampling exploration to obtain reactive trajectories connecting reactive states. (A) In umbrella sampling, a bias potential (*V*_1_, *V*_2_,…, *V*_*n*_) is introduced in the potential energy of the system *U*(*r*), in *N* independent simulations, to achieve exploration of the conformational space across the reaction coordinate, *r*; the corresponding free energy, *F*(*r*), is then obtained through a reweighting scheme (*e.g.*, weighted histogram analysis). In metadynamics, the total bias *V*(*r*, *t*) added to the potential energy *U*(*r*) increases as the simulation progresses. The added bias at instant *t*_i_ is a Gaussian-shaped bias centred at position *r*(*t*_i_) along the reaction coordinate *r*, and the corresponding bias *V*(*r*, *t*_i_) is built by deposition of all the Gaussian-shaped biases introduced until then. Once equilibrium is reached, the cumulative bias *V*(*r*, *t*) can be used to retrieve the underlying free energy *F*(*r*) using a free energy reweighting scheme. (B) In transition path sampling, trajectories are started from the position *r*_i_ along a tentative reactive trajectory with modified momenta in the forward (*p*_i_) and reverse (−*p*_i_) directions, being evaluated as “accepted” or “rejected” based on whether they can connect states A and B (two hypothetical reaction minima). The reaction coordinate can be approximately defined by estimating the committor function between states A and B along tentative reaction coordinates, *r*, *s*, …. Using transition interface sampling, the same probability can be quantified for different slabs *λ*_1_, *λ*_2_, …*λ*_*N*_ along the reaction coordinate, *r*, to reproduce the corresponding sigmoid shape of the committor function of the process, *q*, for which the probabilities at the end states *q*_A_ and *q*_B_ are 0 and 1, respectively, and that at the TS *q*^TS^ is 0.5.

The most common strategy when studying enzyme reaction mechanisms is to add a bias potential to the PES, in this way, damping free-energy differences between low and high energy regions (Fig. 5A). This way, barrier crossings are accelerated within accessible simulation times, enabling efficient sampling of reactions that would otherwise occur only beyond the microsecond timescale.^364,365^ Although kinetic quantities cannot be directly retrieved from these simulations, the resulting biased MD trajectories can, in many cases, be reweighted to reconstruct the underlying unbiased free-energy landscape accurately.

An alternative framework is that of path sampling approaches (Fig. 5B), such as transition path sampling (TPS), which have been widely applied, especially when the exploration of multiple chemical pathways connecting reactants and products as well as their corresponding kinetics are of interest.^366^ These approaches require prior knowledge of the reaction end states and a trial trajectory connecting them to explore both the configurational and momenta space of the reaction. This is done by assessing the ability of different starting conformations to generate reactive trajectories (those connecting reactant and product states) *via* perturbation of the momentum of every atom in the system, which ensures conservation of the shape of the free-energy landscape and of its natural dynamics. As the natural dynamics of the system is conserved, kinetic and thermodynamic quantities can be directly calculated by resorting to the transition interface sampling (TIS) variant, assuming sufficient sampling of the reaction path.^367–370^ Mechanistic information and the reaction coordinate can also be determined after calculation of the committor function, *q*(**x**), for different combinations of geometrical descriptors (*e.g.*, distances, angles, dihedrals, *etc*.).^344,367^ The committor function is the probability that a trajectory initiated from configuration **x** will reach state B before returning to state A (Fig. 5B), which in the context of a chemical reaction informs on how likely a given conformation is to commit to products rather than relaxing back to reactants. Consequently, *q*(**x**) is a sigmoid-like function that takes the values 0 and 1 in the reactant and product states, respectively, and increases steeply through the transition region where *q*(**x**) = 0.5, features for which the committor is often regarded as an ideal reaction coordinate.

However, proper sampling for accurate kinetic and thermodynamic determinations using path sampling typically requires several hundreds of reactive trajectories (especially for reactions exhibiting high barriers), and it also relies on generating suitable initial conditions, namely a set of configurations to launch the reactive paths. This makes such approaches more computationally demanding and their implementation more laborious than bias-based enhanced sampling methods.^366^ As such, we will address the latter with greater detail in this section.

Among the bias-based methods, umbrella sampling and metadynamics are among the most used enhanced sampling approaches for investigating enzyme reaction mechanisms.^341,343,345,346,365,371–375^ These enhanced sampling approaches modify the effective potential energy of the system by adding an external bias dependent on selected collective variables (CVs), which are chosen to describe the slow modes relevant to the chemical event under study. The definition of optimal CVs is an important step to ensure that enhanced sampling simulations efficiently cover the conformational and reactive space associated with chemical reactions, within feasible simulation timescales,^336,376,377^ and the choice and functional form of these CVs are thus among the most critical features in the study of enzyme reaction mechanisms, because an inappropriate choice of CVs can introduce systematic errors and distort the underlying free-energy landscape.^378^ In the study of enzyme reaction mechanisms, CVs are typically based on geometric descriptors associated with bond breaking and formation, namely interatomic distances or atom coordination numbers,^377,379,380^ but more complex CVs might be required to unveil more intricate and collective phenomena, as is often the case in enzyme reactions.^376,381–384^

Umbrella sampling relies on a set of parallel independent MD simulations in which a harmonic potential bias is centred at different values of CV, ranging from reactants to products. By tuning the force constant of the harmonic bias and ensuring CV overlapping across all trajectories, a potential of mean force can be calculated by reweighting the sampled distributions using established schemes such as the weighted histogram analysis method (WHAM),^385^ from which the corresponding reaction free-energy profile can be retrieved.^386^ The implementation of umbrella sampling simulations can be laborious and computationally demanding, because the harmonic bias for each simulation must be calibrated for proper sampling over the CV space by trial-and-error, and sampling over multiple CVs can be inefficient when a reaction coordinate is not well-known beforehand.^362,387,388^

As such, umbrella sampling simulations are particularly more efficient at exploring the conformational space of a reaction once its intrinsic reaction coordinate can be described as a one-dimensional CV. To this end, chain-of-state methods are popular approaches to determine minimum free energy paths and their corresponding intrinsic reaction coordinate with a lower computational cost.^365^ These methods can identify minimum free energy paths by optimizing a string connecting a set of equidistant conformations (also called nodes) connecting reactant and product states. In static approaches, as is the case for the nudged elastic band (NEB) method, these paths are calculated after minimization of the potential energy gradient of each node with respect to their position in the reaction path.^389^ On the other hand, MD-based approaches, including the adaptive string method (ASM) or the finite-temperature string method (FTSM), can explore multiple CVs simultaneously and provide an optimal reaction path by minimizing the forces orthogonal to the direction of the string for the selected CV subset.^390,391^

Alternatively, metadynamics consists of a single simulation where the bias is constructed as a sum of repulsive Gaussian functions deposited on the fly along the trajectory in CV space. Over time, this history-dependent bias discourages revisiting previously sampled configurations, effectively flattening the free-energy landscape. In the long-time limit (and assuming adequate CVs), the bias promotes an approximately uniform exploration of the CV space, and the accumulated bias can be used to reconstruct the underlying free-energy profile up to an additive constant.^305,392^

In principle, metadynamics could overcome the limitations of umbrella sampling because the history-based bias prevents redundant sampling and the resulting smoothened distribution facilitates transitions across barriers. However, it can also lead to overfilling of basins and suboptimal convergence, especially in high-dimensional or noisy CV spaces, as is the case for enzyme-catalysed reactions. Practically, metadynamics is often combined with kinetic control approaches, such as well-tempered biasing^392^ and parallel-tempering,^393^ to overcome the formation of dead-end flat-bottomed potential regions and promote better sampling along the selected CV.^392,394^ These enhanced sampling approaches are quite popular because they result in a smoother sampling behaviour, improved stability and more accurate reconstruction of the underlying free energetics, particularly in complex systems such as enzymes. They also enable the reconstruction of the original Boltzmann equilibrium distribution of the system from relatively short simulations, do not require complex parameterization, and can be implemented relatively easily across different types of systems.

More recently, the conceptual framework of metadynamics was revised to develop the on-the-fly probability enhanced sampling (OPES) approach. OPES generalizes the idea behind well-tempered metadynamics by constructing a bias potential aimed at driving sampling towards a predefined target distribution.

**Fig. 6:**
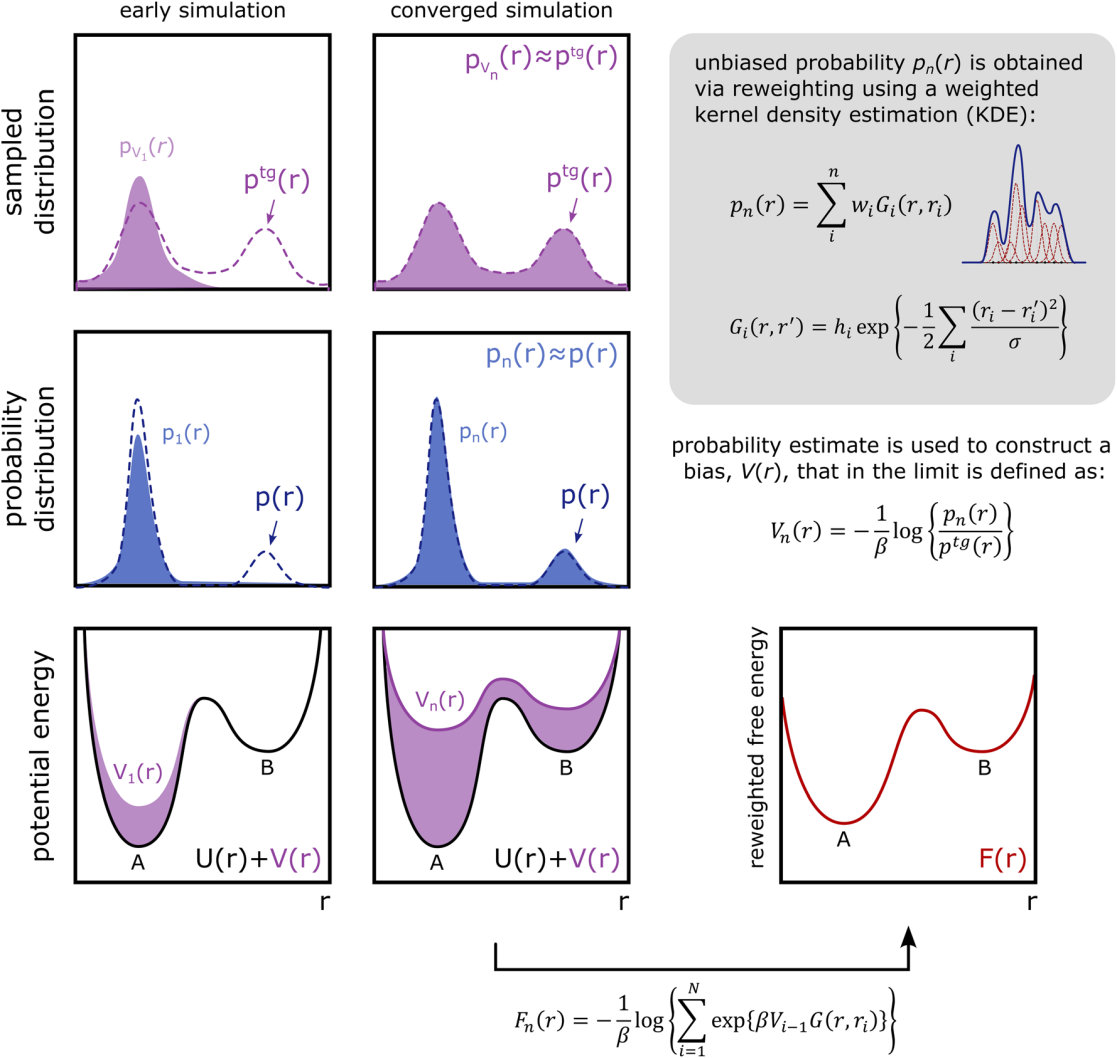
Schematic representation of the on-the-fly enhanced probability (OPES) method. In OPES, the probability distribution at iteration *n*, *p*_*n*_(*r*), is described on-the-fly by combining weighted Gaussian kernels *G*_i_(*r*, *r*′), with normalized weights *w*_i_. By defining a target probability *p*^tg^, generally, a well-tempered form of the equilibrium probability distribution, *p*(*r*), the corresponding probability-dependent bias *V*_*n*_ at iteration *n* is sequentially added to the potential energy of the system *U*(*r*), until the equilibrium probability distribution is obtained (meaning the target probability distribution is sampled). The underlying free energy of the process, *F*(*r*), can then be obtained by a reweighting scheme, as is commonly employed in bias-based enhanced sampling methods. Adapted with permission from E. Trizio, A. Rizzi, P. M. Piaggi, M. Invernizzi and L. Bonati, *arXiv* preprint, arXiv:2410.18019, 2024 (Copyright 2024 by the authors).

Rather than building the bias through simple Gaussian deposition, at each step *n*, OPES estimates *via* reweighting the probability distribution, *p*_n_(*r*), along the CV space, *r*, in real time and constructs a bias that drives it closer to the desired target distribution, *p*^tg^(*r*) (Fig. 6).^395,396^ OPES has shown excellent promise in reducing the risk of suboptimal CV description and producing converged free-energy profiles faster than conventional metadynamics approaches.^396^ The flexibility of the OPES framework has also given rise to several variants summarized by Trizio *et al.*,^397^ each tailored to specific sampling goals, namely for reaction discovery using suboptimal CVs with OPES-Explore,^364,395,398^ for accurate estimation of transition state ensemble (TSE) statistics and rate constants with OPES-Flooding,^381,399^ or for the rigorous calculation of binding and activation free energies with OPES-Expanded.^400–402^

Very recently, Parrinello and coworkers proposed a new enhanced sampling strategy, based on the committor function, which enables extensive sampling of the TSE.^403,404^ They then proposed to integrate this TS-oriented bias with OPES, using the committor as the CV, thus obtaining a framework that enables uniform and efficient sampling of reactive pathways with a greater focus on the TS than in conventional approaches. As this method is based on a broader procedure to machine learning the committor, it will be discussed in more detail in the following section.

On a final note, a holistic study of reaction mechanisms by enzymes involves not only the chemical step but also enzyme activation or preorganisation, substrate/cofactor binding, and product release. In this context, enhanced sampling has proven to be invaluable for capturing substrate binding dynamics and transitions from non-reactive to catalytically competent conformations within enzyme active sites,^377,381,405^ steps that are crucial for developing a fully integrated model of enzyme catalysis that includes both its associated thermodynamics and kinetics.

## 5 Machine learning in the study of enzymatic reaction mechanisms

Throughout this review, we have discussed the intricacies involved in the study of enzymatic reaction mechanisms, from how the initial enzyme model can impact mechanistic studies to how multiple pathways connecting reaction intermediates can complicate the identification of the most probable mechanism. Ultimately, the accurate determination of enzyme reaction mechanisms remains limited by the high computational cost of the electronic structure methods required for reliable quantum chemical accuracy, and by the extensive sampling required to capture rare events that take place during the enzymatic reaction and determine the kinetic and thermodynamic quantities that inform the feasibility of the proposed mechanisms.

The importance of data for informed scientific interpretation has been highlighted several times. We also addressed how data have been of great use in improving our ability to develop tools with improved predictive power and reduced computational cost, *e.g.*, the groundbreaking success of AlphaFold in predicting protein structures from amino acid sequences,^69^ or the ability of EzMechanism to generate mechanistic hypotheses based on reactivity rules derived from the Mechanism and Catalytic Site Atlas (M-CSA).^234^ As AI-based solutions become widespread in almost every scientific field, recent years have witnessed increased interest in the exploration of ML techniques to address challenging questions in chemical reactivity. These range from the development of more efficient methods to explore the landscape of chemical reactions^406^ to novel methods that improve the accuracy of molecular property calculations.^407^ These advances are now beginning to influence the study of enzymatic reaction mechanisms, providing new strategies to balance the computational cost, sampling efficiency, and quantum chemical accuracy.

When it comes to enzymatic reaction mechanisms, two main strategies have been considered to address the long-standing accuracy–efficiency trade-off hampering their computational study: (i) the development of ML-based potentials with the ability to simulate enzyme reactivity over longer timescales while improving energetic and thermodynamic accuracy and (ii) the development of optimal CVs to improve the sampling efficiency of CV-based enhanced sampling simulations.

In this context, traditional QM/MM approaches provide a rigorous physical description of enzymatic reactions by treating the reactive region with QM while modelling the surrounding environment with MM. However, their high computational cost often limits the extent of conformational sampling that can be achieved. ML/MM approaches aim to overcome this limitation by replacing the QM region with MLIPs trained on quantum chemical data, allowing significantly faster simulations and broader sampling. However, their accuracy ultimately depends on the quality and transferability of the training data.

### 5.1 Machine learning potentials for the study of reaction mechanisms

**Fig. 7:**
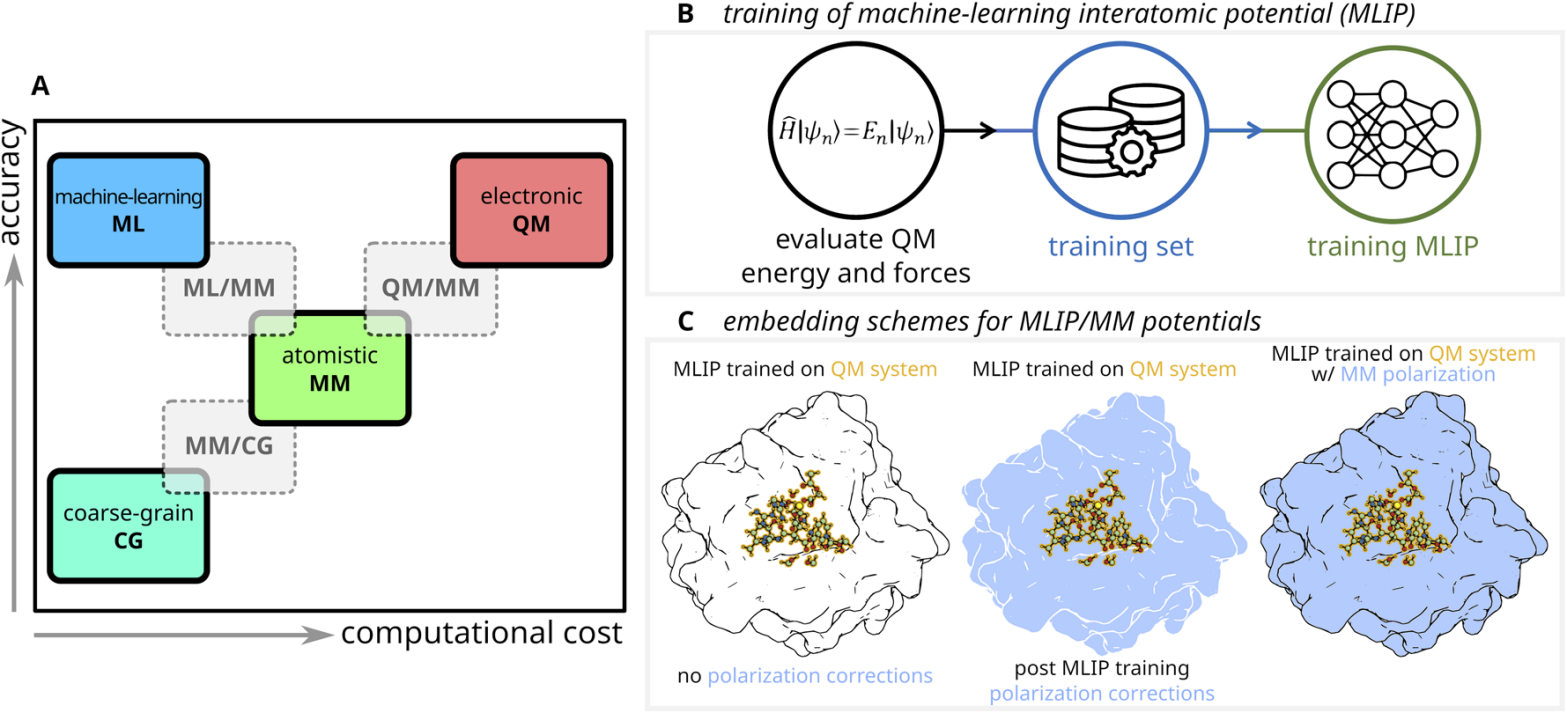
Schematic representation of accuracy–computational cost trade-off for different representations of the potential energy of molecular systems (A); workflow for the training of a machine learning interatomic potential, MLIP (B); representation of different embedding schemes for hybrid MLIP/MM potentials—from left to right, mechanical embedding, polarization-corrected mechanical embedding, and environment-integrated embedding (C).

Promising developments in MLIPs are further reducing simulation costs while retaining near *ab initio* accuracy (Fig. 7A). The conceptual foundation of modern MLIPs dates back to the seminal work of Behler and Parrinello, who introduced high-dimensional neural networks to approximate PESs of atomistic systems, enabling fast evaluation of energies and forces.^408^ In this framework, the total energy is expressed as a sum of atomic contributions, each predicted by an element-specific neural network. The inputs to these networks are local atomic descriptors constructed from atom-centred symmetry functions, which enforce translational, rotational, and permutational invariance.

Since this pioneering work, a wide variety of MLIPs have been developed, such as DeepMD,^409,410^ ANI,^411^ and PhysNet,^412^ as well as more recent equivariant graph neural network architectures such as NequIP,^413^ and MACE, which systematically improve accuracy by encoding many-body and directional information.

These models are trained on large datasets of *ab initio* energies and forces and are capable of DFT accuracy at a computational cost comparable to classical force fields (Fig. 7B). As a result, MLIPs have become powerful tools for MD simulations, enabling extensive sampling of reaction pathways, free-energy landscapes, and conformational ensembles that would otherwise be prohibitive using direct QM methods.

However, despite their remarkable performance, MLIPs remain challenging to apply directly to large heterogeneous systems such as enzymes and solvated biomolecules, since they often rely on local descriptors that limit their ability to capture long-range interactions. To overcome these limitations, hybrid machine learning/molecular mechanics (ML/MM) approaches have emerged as a natural evolution of traditional QM/MM schemes, in which the quantum region is replaced by an ML potential, while the environment is treated with classical force fields.^287^

As in QM/MM schemes, the total energy in ML/MM formulations is commonly decomposed into three terms: the energy of the ML region, the energy of the MM region, and their interaction energy (Fig. 7C). Early implementations adopted a mechanical embedding (ME) strategy, in which the ML potential was evaluated *in vacuo*, and the interaction with the MM environment was described using fixed atomic charges and van der Waals terms.^287^ While computationally efficient, this approximation neglects polarization effects induced by the environment.

To improve upon this limitation, more advanced embedding schemes have been proposed. For example, Semelak *et al.* introduced a polarization-corrected mechanical embedding scheme in which geometry-dependent minimal basis iterative stockholder (MBIS) atomic charges are predicted on-the-fly by a neural network and coupled to the MM environment through an induced-dipole polarization model.^414^ The developed approach incorporated explicit polarization corrections, resulting in good agreement with reference QM/MM electrostatic embedding schemes, and it was successfully applied to aqueous-phase simulations, including solvation structure analysis, vibrational spectroscopy, and torsional free-energy profiles of small molecules, as well as to protein–ligand binding studies, demonstrating quantitative agreement with QM/MM reference data.

An alternative coupling strategy is environment-integrated embedding (EIE), in which the ML potential is explicitly trained in the presence of the MM environment, thereby mimicking full electrostatic embedding in QM/MM. In this approach, information about the surrounding classical region (such as point charges, external electric fields, or full MM configurations) is incorporated directly into the ML model inputs, allowing the network to learn how the environment modulates the PES of the reactive region. As a result, polarization and environmental effects are implicitly captured within the ML energy term, rather than being added as *post hoc* corrections. While EIE schemes can achieve accuracy comparable to QM/MM electrostatic embedding, their practical applicability is limited by the need for specific training datasets generated at the QM/MM level, which substantially increases data requirements and restricts transferability to new systems. Consequently, although highly accurate, EIE models are typically system-specific and less generalizable than ME or PCME approaches. A more complete overview can be found in this review by Grassano *et al.*^287^

Recent applications of ML/MM have demonstrated its potential to model complex systems. In one of the earliest studies, Lahey *et al.* introduced a mechanical embedding scheme for protein–ligand binding, treating the ligand with an MLIP while describing the surrounding protein and solvent using classical force fields.^415^ Their work focused on a set of eight protein–ligand complexes, evaluating both ligand pose prediction and the conformational component of binding free energies. Notably, ML/MM simulations produced ligand conformations in significantly better agreement with crystallographic electron density than those obtained using MM-only models. In addition to improved accuracy, the method demonstrated practical performance, achieving sampling rates of ∼3–4 ns per day for a 69-atom ML region, highlighting its feasibility for routine integration into enhanced sampling workflows.

Beyond binding studies, ML/MM has also been applied to reactive condensed-phase systems, where accurate treatment of polarization and solvent response is critical. Töpfer^416^ and Salehi *et al.*^417^ investigated double proton transfer in the formic acid dimer and the IR spectra of solvated phenols using a mechanical embedding scheme enhanced with geometry-dependent atomic charges predicted on-the-fly by a neural network trained on energies, forces, and dipole moments. For the formic acid dimer, the authors computed free-energy profiles for double proton transfer, solvent radial distribution functions, and hydrogen-bond charge transfer, capturing key features of the system across a broad temperature range.^416^ The same ML/MM approach was applied to phenol and 4-fluorophenol in aqueous solution, where it successfully reproduced the solvent-induced red shift of the OH stretching frequency.^417^ However, it failed to capture the fluorination-dependent blue shift, which was correctly described by QM/MM calculations. This limitation points to the difficulty of modelling solute–solvent polarization effects using mechanical embedding alone.

Furthermore, ML/MM approaches have shown particular promise in the study of metalloenzymes, where traditional force fields often struggle to describe metal coordination accurately. Xu *et al.* implemented a mechanical embedding scheme in which the metal-binding group was treated with an ML potential, while the remainder of the protein retained a classical description.^418^ The model was trained on energies, forces, and RESP charges, and it reproduced Zn–ligand bond lengths and angles and captured fluctuating Zn^2+^ charges reflecting learned polarization effects.

In a related study, Ding *et al.* developed an ML/MM approach for the same class of systems using the DeepPot-SE framework.^419^ Their setup improved structural accuracy, correcting spurious over-coordination of Zn^2+^ by water observed in MM-only simulations and yielding Zn–S distances in better agreement with crystallographic data. Reported performance ranged from 0.6 to 1.7 ns per day, demonstrating the feasibility of this approach for extended simulations of metalloproteins.

More recently, Ohmura *et al.* introduced a GPU-accelerated ML/MM toolkit for characterizing enzymatic reaction mechanisms, combining the Universal Model for Atoms (UMA) potential with automated TS searches and Hessian evaluation.^420^ Applied to chorismate mutase, the method reproduced activation barriers consistent with QM/MM and experimental data, while achieving full reaction path characterization within hours on a single GPU.

Despite the favourable computational scaling of MLIPs during production simulations, ML/MM approaches can shift a substantial part of the cost from the simulation itself to the construction, validation, and deployment of the ML potential. This aspect is particularly relevant when generating reactive potentials for enzymatic mechanisms, where the training data must cover reactants, intermediates, products, transition-state-like structures, and off-equilibrium geometries. In practice, this often requires several active learning cycles where configurations poorly described by the current ML potential are identified, recalculated at the reference level of theory, and added to the training set to progressively improve the model.

Strategies that integrate enhanced sampling within the active learning workflow can help collect transition-state geometries and improve the description of poorly sampled reactive regions.^421,422^

Overall, these developments establish ML/MM as a scalable, accurate, and increasingly versatile alternative to traditional QM/MM, offering a data-driven route toward next-generation multiscale modelling in enzyme catalysis.

### 5.2 From chemical intuition-based to machine learning collective variables

Although CV-based approaches differ in their specific implementations, they share a common conceptual framework: (i) identify a reduced set of CVs that capture the slow degrees of freedom governing rare events, (ii) construct a bias potential that enhances exploration along these CVs, and (iii) ensure that the bias converges in a statistically controlled way such that the underlying free-energy surface can be reconstructed.

The definition of optimal CVs is a particularly important step to ensure that enhanced sampling simulations efficiently cover the conformational and reactive space associated with chemical reactions on feasible simulation timescales.^336,376,377^ The construction of suitable CVs is often nontrivial because enzyme reactions often involve complex structural effects that are hard to capture by a low-dimensional set of CVs. Poorly chosen CVs may lead to ineffective bias and spurious results when the CV is orthogonal to the true reaction coordinate, as well as failing to capture the complex, collective, and sometimes non-local rearrangements that underlie enzymatic function.

Data-driven methods aim to address this limitation by identifying low-dimensional representations from high-dimensional MD data.^406^ These approaches generally follow a two-step procedure: (i) descriptor selection, where a physically motivated set of molecular descriptors (*e.g.*, distances, angles, coordination numbers, *etc*.) is defined to describe the system, and (ii) dimensionality reduction or learning, where these descriptors are projected into a reduced space using statistical or ML techniques, yielding one or more CVs that monitor slow structural transitions between long-lived states.

Most simple data-driven CVs include the well-known principal component analysis (PCA), which aims at identifying CVs that can maximally encode the variance of the training data and proved useful in establishing dynamic connections between enzyme collective motions and their catalytic activity,^423,424^ or harmonic linear discriminant analysis (HLDA), in which the resulting CV is a linear combination of descriptors optimized to distinguish user-defined reaction end states.^382^

Linear approaches like HLDA are interpretable and computationally efficient, but they are inherently limited in their ability to capture the non-linear and multidimensional features that often govern enzymatic reactions. In many biochemical systems, the relevant slow modes involve coupled motions of side chains, substrate rearrangement, or water molecules, which are not well described by any single geometric descriptor or a simple linear combination thereof. To address these limitations, a growing class of methods leverages the power of ML to learn non-linear CVs directly from simulation data. These ML-based approaches offer enhanced flexibility while remaining compatible with enhanced sampling techniques such as metadynamics or OPES.

#### 5.2.1 Machine learning collective variables

In the framework of ML CVs, the CV is expressed as the output of an ML model, typically a feed-forward neural network.^406^ The output CV is a differentiable non-linear function that maps each molecular conformation to a value in the CV space. This learned CV can then be used in enhanced sampling simulations to promote exploration of the desired reaction and ultimately recover thermodynamic observables (*e.g.*, free energies) *via* standard reweighting.

**Fig. 8:**
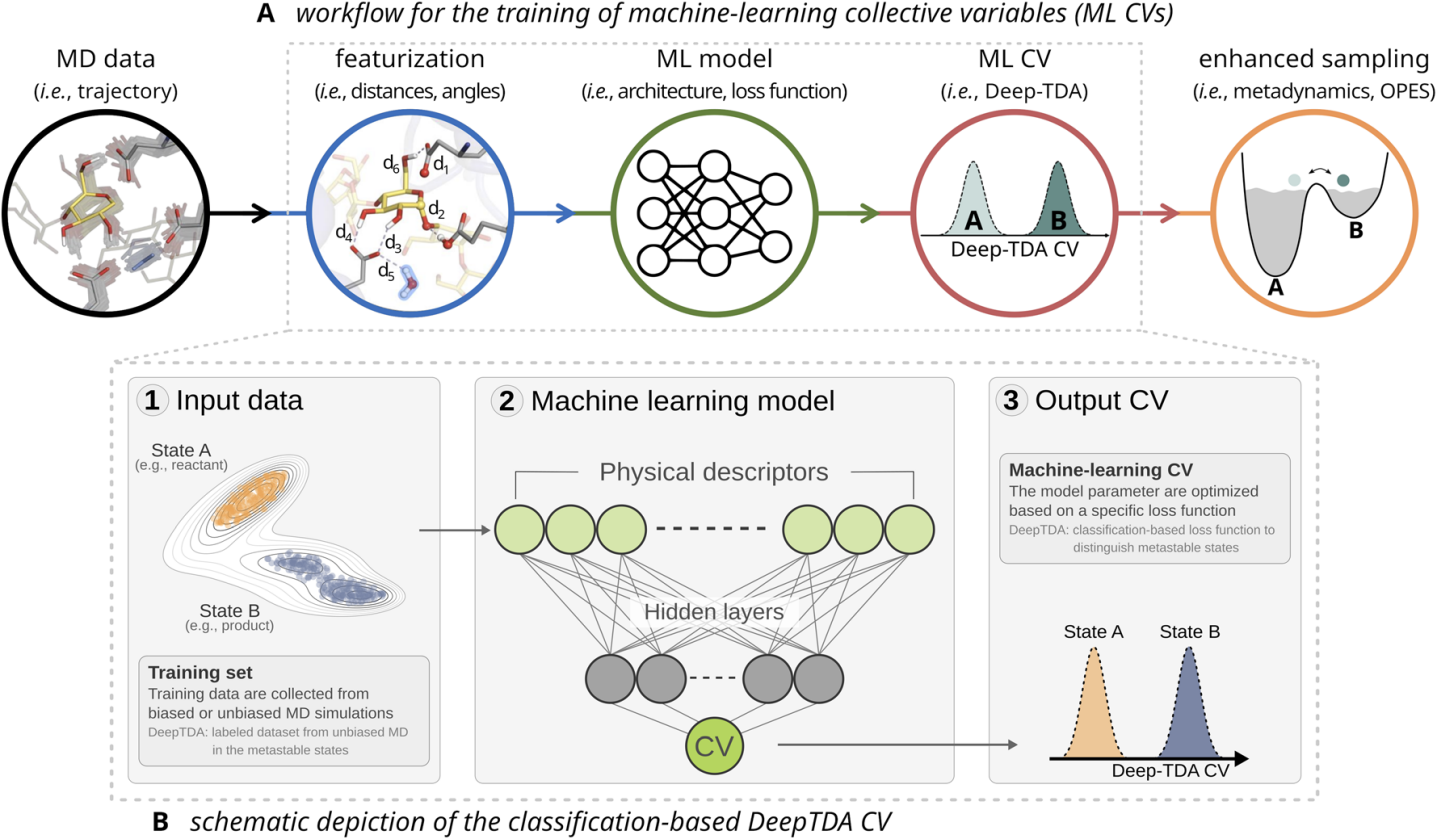
Workflow for the training of machine learning collective variables, ML CVs (A); the classification-based DeepTDA CV is schematically depicted as an example (B): (1) the training data are obtained from MD simulations, representing the system through a set of physical descriptors; (2) these data are the input of a neural network, whose output represents the CV; (3) optimization of the model parameters is performed based on a specific learning criterion, according to the chosen method. Panel B adapted from ref. 425 (Copyright 2023 American Institute of Physics) and ref. 426 (Copyright 2021 American Chemical Society).

Most ML CV development follows a common methodological structure, typically consisting of three stages (Fig. 8A): (i) descriptor construction and data generation, (ii) model learning and optimization, and (iii) deployment and enhanced sampling.

Specifically, the inputs of ML CV models are typically simple, but may account for many physical descriptors (*e.g.*, distances, angles, coordination numbers, contact maps, *etc*.) encoding information about the chemistry, geometry and symmetry of the system. Once this set of input descriptors is defined, representative data are gathered from existing simulations, either unbiased trajectories or enhanced sampling runs, *e.g.*, structures from different ligand-binding poses^57,377^ or catalytic states.^376^ These data form the training set used for the optimization of the neural network. The optimization is carried out by minimizing a suitable loss function, which formalizes the learning criterion characterizing the CV itself (*e.g.*, a classification approach, Fig. 8B). Notably, there is a strong interconnection between the required type of data and the learning criterion to be used for neural network optimization.

A complete overview of ML-based CVs has been recently reported by Trizio and Bonati.^406^ These approaches differ primarily in the type of information they extract from simulation data, depending on whether the goal is distinguishing predefined states – Deep Linear Discriminant Analysis (DeepLDA)^427^ and Deep Targeted Discriminant Analysis (DeepTDA),^426^ – or identifying slow collective motions – Deep Time-lagged Independent Component Analysis (DeepTICA).^428^

In more detail, DeepLDA builds upon the traditional HLDA framework by first learning an optimal non-linear combination of input descriptors on which linear discriminant analysis is applied more effectively. In practice, given a set of descriptors and corresponding predefined macrostates (*e.g.*, reactant and product), the neural network is trained to find a CV space in which the states are well separated.^427^ This is achieved by optimizing a loss function inspired by LDA that penalizes intra-class variance and rewards inter-class separation, thereby emphasizing differences between macrostates. A representative application of DeepLDA is found in the study of calixarene host–guest systems, where a DeepLDA CV describing water fluctuations was used to accelerate binding and unbinding and compute free energies.^429^ The CV not only enabled efficient exploration of the binding free-energy landscape but, through sensitivity analysis, revealed how solvent rearrangements and water exclusion from the binding pocket were key features controlling the binding event, a level of mechanistic insight that would have been difficult to obtain with simple geometric descriptors.

DeepTDA builds upon the discriminative philosophy of DeepLDA but introduces a more flexible and general framework in which each metastable state (*e.g.*, reactant, intermediate, or product) is associated with a predefined target distribution in the CV space.^426^ The neural network is then trained so that the population of structures assigned to each state matches when projected in the CV space. This is achieved by minimizing a loss function that quantifies the dissimilarity between the empirical distributions in each state and the predefined target distributions (Fig. 8B). By specifying target distributions directly, DeepTDA allows greater control over the shape and resolution of the learned CVs and provides a simpler framework to set up, especially when multiple states are involved. For example, one can choose a target set of well-separated Gaussian distributions, each associated with a state, thereby encouraging sharp discrimination between states, possibly reflecting hierarchical transitions and multiple intermediate processes. This flexibility in target design makes DeepTDA especially suitable for complex reaction pathways involving more than two relevant metastable states or non-sequential transition pathways.^426^

As for DeepLDA with LDA, Deep-TICA is a non-linear extension of the time-lagged independent component analysis (TICA), sharing the same idea of learning a latent space where TICA is more effective. However, unlike approaches like DeepLDA and DeepTDA, which aim at distinguishing states, the goal of DeepTICA is to identify CVs that are informative about the long-timescale dynamics of molecular systems, namely enzyme binding, allostery or folding events. To this aim, DeepTICA is trained on reactive trajectory data by minimizing a loss function that leads to CVs whose time autocorrelation is maximized, or, in other terms, correspond to the slowest modes of the process.^428^

Collectively, these three methods illustrate how ML and enhanced sampling can be combined to automate the search for meaningful reaction coordinates. The choice among DeepLDA, DeepTDA, or DeepTICA is often dictated by the nature of the system under study, whether the transition involves two states or multiple intermediates, or is best described as a continuous slow rearrangement, and can be further refined through iterative cycles of learning and exploration. As demonstrated in a growing number of applications, including enzyme catalysis,^57,377^ protein folding,^430,431^ and ligand binding,^429,432–434^ these deep-learning approaches are rapidly becoming standard tools in the construction of meaningful CVs for rare event sampling.

In practice, these ML CVs can be trained with Python-based packages such as *mlcolvar*^425^ and then can be used as CVs in PLUMED,^435^ facilitating their practical integration into MD workflows.

#### 5.2.2 Machine learning the committor function

More recently, ML approaches have also opened new avenues for studying rare events by allowing the committor function, *q*(**x**), to be learned directly from simulation data. As we discussed in a previous section, the committor function is a central object in path sampling simulations and in the direct calculation of reaction kinetics, providing a probabilistic and mechanistically agnostic way to define the TSE, conventionally associated with the isosurface where *q*(**x**) = 0.5, which makes it an ideal reaction coordinate for studying chemical events.

However, despite its theoretical appeal, estimating the committor function has traditionally required large numbers of unbiased or transition path trajectories, making it impractical for large systems or reactions with significant barriers such as those encountered in enzymatic catalysis (see Section 4.3).

To overcome this challenge, Parrinello and coworkers^403^ proposed an alternative approach where the committor is machine-learned following a self-consistent iterative procedure, in which cycles of sampling and learning are alternated. In this approach, in the same spirit of the other ML CVs discussed, the committor is represented as a neural network *q*_θ_(**x**), which is then optimized following the Kolmogorov variational principle to which the committor obeys. In practice, this amounts to (i) imposing simple boundary conditions *q*_θ_(**x**_A_) = 0 and *q*_θ_(**x**_B_) = 1, where **x**_A_ and **x**_B_ are configurations belonging to the reactant and product basins, respectively, and (ii) minimizing the so-called Kolmogorov functional:

2 *K*[*q*_θ_(**x**)] = 〈|∇_*u*_*q*_θ_(**x**)|^2^〉_*U*(**x**)_,

where 〈·〉_*U*(**x**)_ denotes the average over the Boltzmann ensemble defined by the interatomic potential *U*(**x**) and ∇_*u*_*q*_θ_(**x**) is the reduced-mass gradient of the committor. As for other deep-learning CVs, each configuration **x** is represented by a set of descriptors used as input features for the neural network.

One of the key innovations in this framework is the introduction of the committor-based Kolmogorov bias potential, given by3
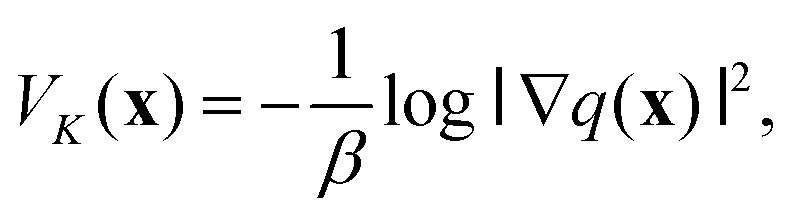


which is more negative in the transition region and close to zero in the stable basins. This potential focuses sampling efforts on the TSE, the region that is generally least accessible in enzymatic simulations. As a consequence, unlike conventional enhanced sampling techniques, the committor-based biasing strategy allows an extensive and balanced sampling of configurations in the TS region, effectively overcoming the challenges associated with sampling critical configurations during rare events, while still accelerating efficient free-energy estimates. This bias has been recently used in combination with the OPES scheme in a study in which Trizio *et al.* used a smoother version of the committor, 
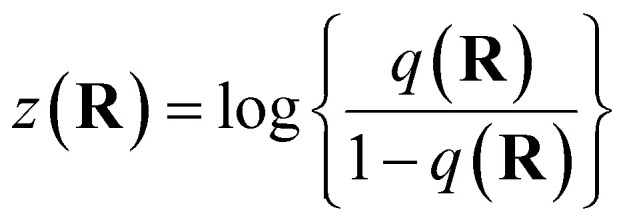
, as a CV in OPES to flatten the free-energy surface and promote transitions between metastable states, while the Kolmogorov bias *V*_*K*_ ensured thorough exploration of the TSE.^404^

**Fig. 9:**
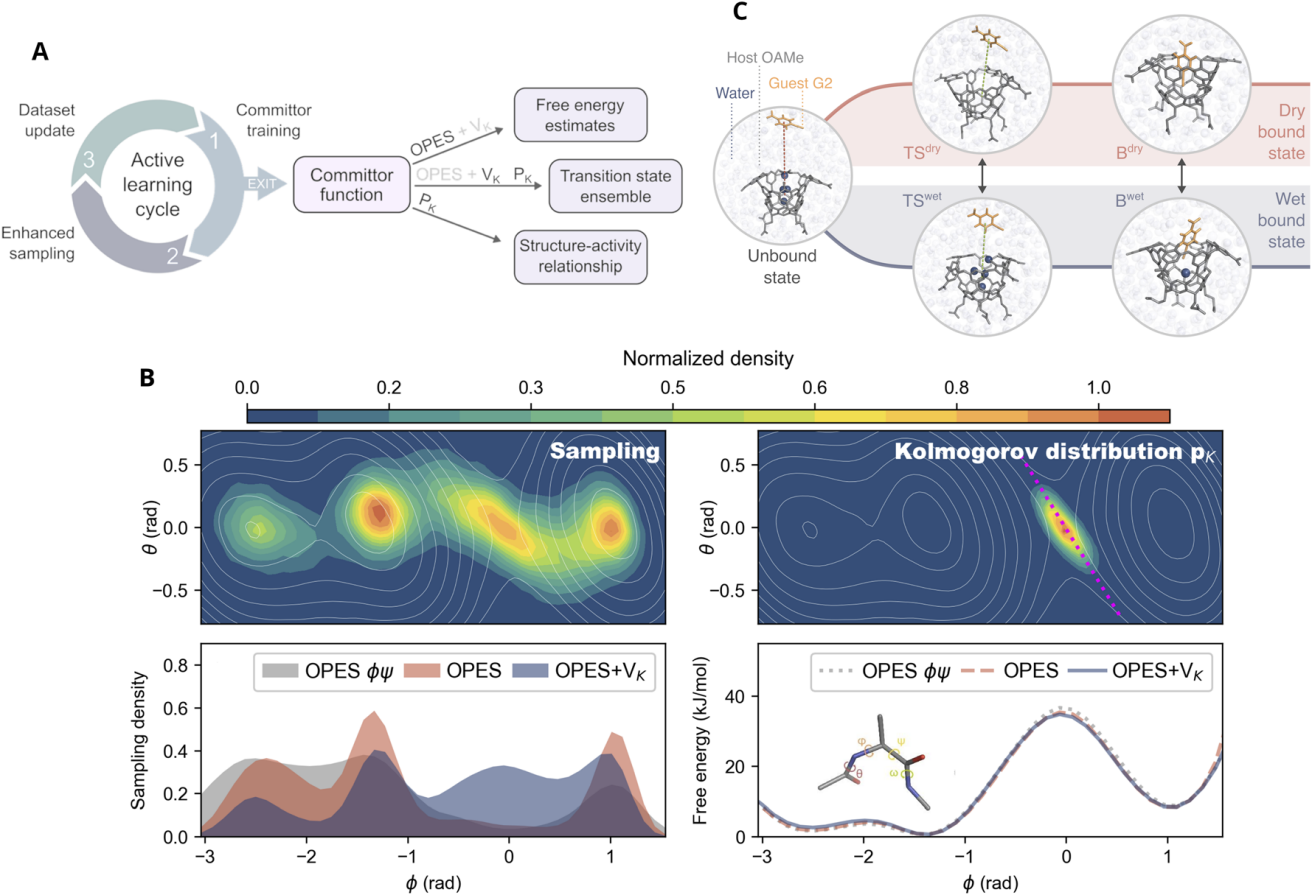
Workflow for the active learning strategy for the committor training under the OPES + *V*_*K*_ scheme and the output information achievable once the OPES + *V*_*K*_ simulations are run using an optimized committor ML CV (A); normalized densities for the *φ* and *θ* torsions of the dialanine peptide under the OPES + *V*_*K*_ scheme and the corresponding Kolmogorov distribution, *p*_k_, the sampling density along the *φ* torsion and the corresponding reweighted free energy (B); molecular representation of metastable states and TSs of the “dry” and “wet” binding kinetics of a G2 ligand guest to an OAMe octa-acid calixarene host, obtained from the clustering analysis of the OPES + *V*_*K*_ simulations with the optimized committor ML CV (C). Panel (A) is reprinted in part with permission from ref. 373. Copyright 2026 American Chemical Society. Panels (B) and (C) are reproduced from ‘Everything everywhere all at once: a probability-based enhanced sampling approach to rare events, E. Trizio, P. L. Kang and M. Parrinello, *Nat. Comp. Sci.*, 5, Springer Nature, 2025’, with permission from SNCSC.

From a practical point of view, learning the committor requires an active learning strategy, in which an initial estimate of the committor obtained from boundary condition data (*e.g.*, unbiased simulations of reaction end states) is progressively refined using data collected from subsequent simulations within the OPES + *V_K_* scheme (Fig. 9A). Nevertheless, although in early iterations the committor model behaves effectively as a classifier distinguishing between the two basins (similarly to Deep-TDA), its accuracy and predictive power rapidly improve as new configurations near the TS region are sampled and fed to the neural network for committor retraining, until it converges to a variationally optimized solution after a few iterations.

A key feature of this approach is its interpretability, as the Kolmogorov bias used to efficiently sample TS regions can also be used to identify which configurations significantly contribute to the TSE based on the Kolmogorov probabilities, *p*_K_(**x**) ∝ |∇*q*(**x**)|^2^exp[*βU*(**x**)] (Fig. 9B). This provides precious insight into the structural and energetic factors governing catalytic pathways and highlights configurations that meaningfully contribute to the reaction rate. Since the committor function captures the true dynamical progression from reactants to products, it also defines a natural reaction coordinate to reconstruct the reaction pathway in a physically interpretable way, providing an excellent tool for detailed mechanistic analysis. For instance, the reaction configuration space can be converted into segments for further configuration analysis by applying, *e.g.*, clustering and featurization techniques. This analysis does not only reveal dominant pathways and competing mechanisms but can also highlight key residues, solvent interactions, or conformational rearrangements that govern the catalytic function.^376,436^

More recently, this methodology has also been applied successfully to complex cases such as peptide conformational changes, protein folding, and ligand binding. In protein folding, for example, the method captured multiple folding routes and side-chain rearrangements critical for forming native contacts. In host–guest binding (Fig. 9C), it distinguished between “dry” and “wet” binding mechanisms and revealed solvent-mediated metastable states that control binding kinetics.^404^ As the field continues to move toward more automated and interpretable workflows^437^ for simulating enzyme catalysis, committor-based sampling is likely to play an increasingly central role in mechanistic enzymology.

#### 5.2.3 Interpretability of machine learning collective variables

A recurring concern with ML-based CVs is loss of interpretability. Unlike linear methods, such as HLDA, which yield an explicit linear combination of descriptors, neural networks may encode complex non-linear functions that are not easily traced back to physical variables. As such, a parallel field of research within the domain of ML CVs concerns the development of strategies to mitigate the loss of interpretability in these CVs. Gradient-based sensitivity analysis and sparse linear surrogate models are two popular strategies in this regard. Gradient-based sensitivity analysis provides a practical way to assess the relevance of each descriptor by averaging the computed absolute gradients of the learned CV with respect to the input features along a representative dataset. This yields a measure of how strongly the CV responds to variations in each descriptor, providing an intuitive ranking of feature importance and revealing whether individual descriptors dominate in different basins or along the transition pathway. This analysis can also be used to guide dimensionality reduction by identifying and retaining only the most relevant descriptors for retraining a simpler or more focused CV. In contrast, sparse linear surrogate models provide an alternative strategy to approximate the non-linear ML CVs using a sparse linear model, such as LASSO regression,^438,439^ or sparse logistic classifiers^440^ trained to reproduce the ML model output from a limited set of inputs. From these surrogate models, it is possible to identify a minimal subset of descriptors whose weighted combination best reproduces the behaviour of the ML CV. Importantly, the resulting sparse coefficients represent a physically interpretable mapping that highlights the essential geometric or chemical features associated with the transition, without requiring explicit inspection of the underlying neural network.

#### 5.2.4 Graph neural networks for descriptor-free collective variables

Despite the attempts to render ML CVs more interpretable, the aforementioned descriptor-based neural network models still rely on a predefined set of features, which can introduce bias or overlook hidden degrees of freedom, *i.e.*, risking missing important interactions. To overcome these limitations and further streamline CV construction, recent developments have turned toward Graph Neural Networks (GNNs),^441^ a class of ML models that can learn directly from the molecular structure, without requiring hand-crafted descriptors.

Indeed, GNNs offer a fundamentally different approach by representing molecules as graphs, where atoms are represented as graph nodes and the edges connecting them encode spatial and chemical relationships. By learning directly from these molecular graphs, GNNs can process raw structural data without requiring an explicit definition of descriptors, thus bypassing the manual feature engineering bottleneck. Another key advantage of some GNN architectures is their ability to respect rotational, translational, and permutational invariance properties. Through message-passing schemes, GNNs iteratively update atomic representations based on their local environments, capturing both short- and long-range interactions in a natural and data-driven manner. This structural awareness allows GNNs to identify subtle patterns associated with rare events that might be difficult to encode through fixed geometric descriptors alone. Despite their black-box nature, ongoing research is making GNNs increasingly interpretable. For example, techniques such as attention mechanisms or feature attribution^442^ can highlight which atoms or regions of the molecule contribute most to the learned CV. Additionally, once a GNN-based CV is trained, its output can also be approximated with a sparse linear surrogate model, allowing the extraction of the most influential chemical features in a physically transparent form. This combination of expressive power, minimal reliance on human intuition, and growing interpretability tools positions GNNs as a highly promising frontier in enhanced sampling of biochemical systems.^442–445^ As computational frameworks evolve, GNNs are likely to play an increasingly central role in the construction of robust, generalizable, and interpretable CVs for complex molecular simulations.

## 6 Case study: α-amylase

The case of α-amylase provides a comprehensive example of how advances in the modelling of enzyme reactions have deepened our understanding of enzyme catalysis. From early single-conformation QM/MM calculations investigating enzyme reaction mechanisms^446^ to multi-conformation QM/MM and QM/MM MD simulations that capture dynamic features of enzyme reactivity and improve the sampling of thermally accessible states,^315,336,379^ we then progressed to the application of ML CVs to better capture slow catalytic motions (beyond the nanosecond timescale) and improve the potential of enhanced sampling methods to enable a better description of enzyme binding and reactivity.^376,377^ Such a comprehensive approach has led to the identification of promising transferable and data-driven patterns of enzyme reactivity,^57^ which we hope may constitute an inspiring example for future research endeavours.

**Fig. 10:**
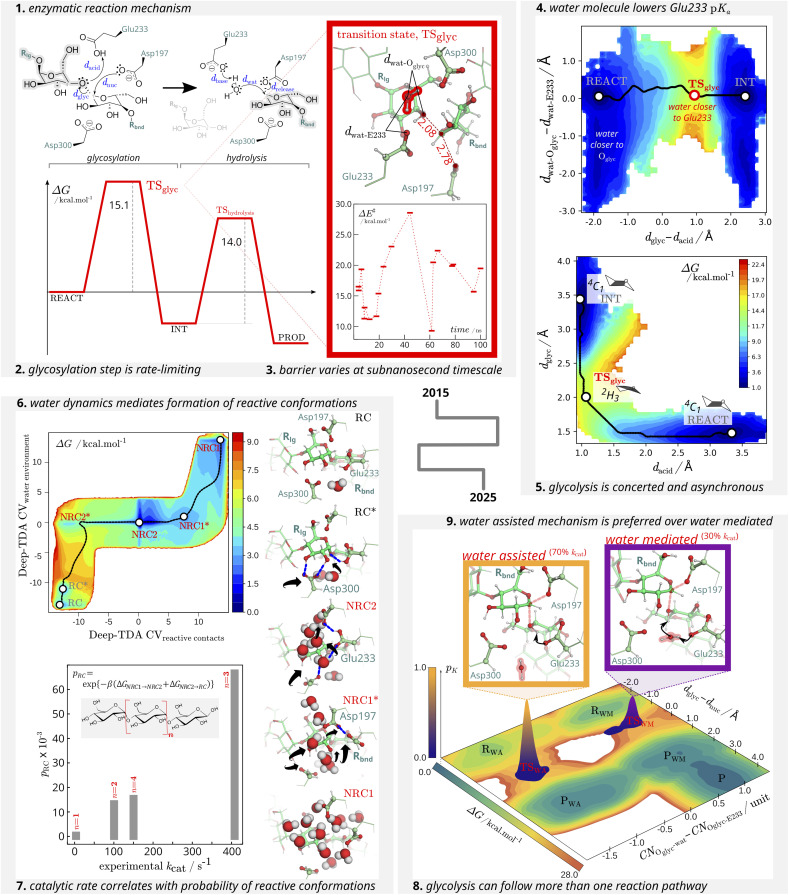
Examples of some of the most representative results obtained computationally in the study of human pancreatic α-amylases, starting with single-PES QM/MM calculations, extending to multi-PES QM/MM and umbrella sampling QM/MM MD simulations, and finally combining ML-trained CVs and enhanced sampling simulations. Using single-PES QM/MM calculations, we determined the reaction mechanism of the enzyme (1) and its rate-limiting step – the glycosylation step (2) –, and using multi-PES QM/MM, we observed that the activation energy of the rate-limiting step varied on a subnanosecond timescale (3) and that a water molecule close to Glu233 could lower its p*K*_a_ and favour catalysis (4). Performing umbrella sampling QM/MM MD simulations on the rate-limiting step of the reaction, we further verified the contribution of water to catalysis (4), confirming that the rate-limiting step followed an asynchronous concerted step (5) and mapping the conformational changes of the reacting sugar and their contribution to catalysis. Using Deep-TDA ML CVs, we were able to assess the influence of water on the formation of reactive enzyme conformations (6), further showing that the formation of reactive conformations contributed to the catalytic rate of the enzyme across substrates of different lengths (7). Finally, we were able to train the committor function of the rate-limiting step and explore it to identify different reaction pathways for the glycosylation (8) and their corresponding contribution to the catalytic rate of the reaction (9). Parts of the figure were adapted with permission from ref. 315 (Copyright 2018 American Chemical Society), ref. 376 (Copyright 2025 American Chemical Society), ref. 377 (Copyright 2023 American Chemical Society), and ref. 379 (under the terms of the CC-BY 4.0 license).

In Fig. 10, we summarise some of the major findings we achieved using physics-based computational methods when studying human pancreatic α-amylase. Since the enzyme is a very important target for the treatment of type 2 diabetes, a fundamental understanding of its substrate binding and catalytic mechanism may pave the way for the rational design of novel drugs against this disease and thus support the challenging efforts of current drug discovery pipelines.

Back in 2015, we published the first computational study on the reaction mechanism of maltopentaose hydrolysis by human pancreatic α-amylase using hybrid QM/MM calculations,^446^ alongside the study of other reaction mechanisms using both QM cluster models and hybrid QM/MM calculations.^446–450^ After modelling a catalytically active enzyme–substrate complex and refining it using MD simulations, we successfully characterised the complete reaction mechanism of maltopentaose hydrolysis based on a representative conformation of the full enzyme–substrate complex. Using the ONIOM scheme with calculations at the DFT/AMBER hybrid level, we confirmed that the reaction mechanism comprised two main steps: (i) glycosylation of a nucleophilic Asp197 located close to the anomeric carbon of the reacting glucosyl unit, preceded by protonation of the glycosidic oxygen of the adjacent sugar by the catalytic acid residue Glu233; (ii) hydrolysis of the glycosylated Asp197 intermediate by a bulk water molecule that becomes nucleophilic upon deprotonation by the basic form of Glu233 through a bridging water molecule.

Although the study was based on a single conformation and therefore did not account for the dynamic nature of the enzyme–substrate conformational landscape, we identified the glycosylation step as rate-limiting with a free-energy barrier of 15.1 kcal mol^−1^ calculated at the B3LYP/6-311++G(2d,2p):AMBER//B3LYP/6-31G(d):AMBER level, remarkably close to the 14.4 kcal mol^−1^ estimated from experiments using TST. In addition, the study showed that activation of the nucleophilic water during the hydrolysis step is facilitated by Glu233 through a second bridging water molecule, providing an alternative to the direct deprotonation of the nucleophilic water by Glu233, as previously reported in the literature.^447–449^

Since enzyme catalysis is a dynamic process, and building on previous studies on enzyme systems identifying a narrow subset of reactive conformations, the near-attack conformation (NAC), in which enzyme catalysis can occur efficiently,^451–453^ we set out to explore the features characterising NACs in α-amylase. In particular, we employed multi-conformation QM/MM (multi-PES) calculations to establish relationships between selected geometric features in the α-amylase active site and the free energy calculated for the rate-limiting glycosylation step.^315^ Our calculations carried out on 19 enzyme–substrate conformations, indicating that the free-energy barrier for the glycosylation step can range from 9 to 28 kcal mol^−1^ between conformations sampled on a sub-nanosecond timescale, revealing that small, fast rearrangements at the active site can significantly influence catalysis.^300,303,314,315^ Together with the proximity of the catalytic acid Glu233 to the glycosidic oxygen and of the nucleophilic Asp197 to the anomeric carbon of the reactive glucosyl unit, we identified a hydrogen bond formed between a labile water molecule and Glu233. A correlation was observed between these distances and the activation barrier, with the distances fluctuating rapidly and continuously during the simulations. That interaction appears responsible for lowering the residue's p*K*_a_, promoting its deprotonation by the glycosidic oxygen and subsequent glycosidic bond cleavage with a lower energy barrier.

To further explore conformational dynamics in the study of reaction mechanisms, we performed umbrella sampling QM/MM MD simulations for four representative conformations derived from the starting X-ray structure, each corresponding to distinct calculated energy barriers for the glycosylation step. This approach aimed to assess how more extensive conformational sampling could impact the mechanistic description of the glycosylation step in α-amylase.^336,379^ Interestingly, we observed that different starting conformations, although extracted from closely spaced time frames, still resulted in distinct free-energy barriers, qualitatively following the same trend observed in the static multi-conformation QM/MM approach (11.0–16.8 kcal mol^−1^*vs.* 9.3–28.1 kcal mol^−1^), albeit within a narrower range. Thus, we concluded that enzyme active site preorganization remains a key factor in the computational study of enzyme reaction mechanisms, regardless of whether adiabatic mapping or short timescale dynamics enhanced sampling techniques are employed, while acknowledging that some conformational requirements affecting catalysis may occur on timescales beyond those captured in the simulations.^336^

We were able to assemble a comprehensive view of the glycosylation mechanism catalysed by α-amylase by integrating data from all these simulations. We calculated a free-energy barrier of 13.9 kcal mol^−1^, in excellent agreement with the experimentally reported value of 14.4 kcal mol^−1^. We also determined free energy contributions from interactions involving specific active site residues, namely Glu233 and Asp197, as well as Asp300, reported to contribute to TS stabilization and the water molecule tuning the p*K*_a_ of Glu233, further complementing our previous work using multi-PES QM/MM adiabatic mapping calculations. In particular, we observed that the combination of critical distances involving Glu233 and Asp197 and the availability of water near the glycosidic oxygen of the cleaved bond are determinant factors that identify productive conformations, whereas the strong hydrogen bond formed between a water molecule and Glu233 is critical for lowering the free-energy barrier at the TS.^379^ We also observed that productive substrate conformations require the reactive glucoside unit to adopt a ^4^C_1_ chair conformation, which undergoes a change to ^2^H_3_ half-chair conformation at the TS, restoring the more stable ^4^C_1_ chair conformation once glycosylation is complete, complementing the extensive literature on carbohydrate conformational dynamics in catalysis by glycosidases.^454–456^

With the growing application of ML to chemical problems, we identified reactive and non-reactive enzyme conformations of α-amylase and calculated their relative free energies and associated kinetic parameters.^381,457^ By using graph-based CVs to classify reactive and non-reactive conformations through QM/MM MD simulations with the on-the-fly probability enhanced sampling (OPES) approach,^396^ we identified the slow collective modes contributing to α-amylase reactivity and used them as descriptors to design machine-learned collective variables (ML CVs). From the latter ML CVs, we outlined the kinetic path connecting non-reactive to reactive (near-attack) conformations based on MD simulations combined with the OPES framework.^377^ Thus, we complemented the available literature on the importance of displacement of water upon substrate binding to activate enzyme catalysis,^458^ by showing that the formation of NACs in α-amylase occurs through a gradual displacement of water molecules from residues Asp197 and Asp300, as the substrate becomes buried within the active site, with the rate-limiting step being the displacement of two water molecules hydrogen-bonded to the reactive glucosyl unit that prevent the interaction with those aspartates. The developed framework was also applied to different substrates with known activities and catalytic rates^459^ without requiring further training of the ML CVs, and we observed a correlation between the experimentally determined *k*_cat_ values and the probability of finding the enzyme–substrate complex in a reactive conformation.^57^

Our results indicated that initial transitions between non-reactive conformations are largely entropy-driven, as the active site becomes increasingly desolvated. Consequently, smaller substrates exhibit the largest free-energy penalties due to their lower desolvation capacity. Conversely, transitions to reactive conformations require tighter substrate binding and rely heavily on the size complementarity between the active site cleft and the substrate. This means that the lowest free-energy penalties occur when the substrate fits the enzyme's binding cleft most closely. These observations could be highly relevant for guiding both drug design strategies and the engineering of enzyme active sites that better accommodate selected substrates of interest, and the generalization of our scheme to other enzyme classes is therefore highly desirable. Indeed, this framework has already been successfully combined with QM/MM simulations using the OPES flooding approach, to show that the experimentally measured *k*_cat_ values of chorismate mutase and α-amylase can be better explained when both the formation of reactive conformations and the chemical catalytic step are considered.^381^

In our latest work on α-amylase, we also characterised TSEs corresponding to two possible reaction pathways for the glycosylation step and the associated free energies.^376^ We took advantage of both the potential of ML and a rigorous physical interpretation of the committor function^403^ to train an ML model approximating the committor function for the glycosylation step of α-amylase, which we then explored using the OPES approach, as described in the previous section. By using the committor function as an ML CV within OPES, we achieved improved sampling of the TS region, which is generally undersampled due to its transient nature, thereby enabling the calculation of TSEs corresponding to various reaction pathways for the glycosylation step of α-amylase.

We found that the glycosylation proceeds through either a water-mediated or a water-assisted pathway, depending on whether a water molecule bridges the proton transfer from Glu233 to the glycosidic oxygen or facilitates this transfer by modulating the acidity of Glu233. The water-assisted mechanism proved more kinetically favoured (70% *vs.* 30%), in line with our previous work,^315,379,446^ although the thermodynamically more stable product state corresponds to formation *via* the water-mediated mechanism. Analysis of the calculated TSEs once again confirmed the previously reported crucial role of water in modulating the acidity of Glu233.^315,379^ Both TSEs exhibited very similar structural features, except for the interaction between Glu233 and water, with the highest TS probabilities corresponding to configurations featuring the combination of short hydrogen bond distances and optimal angles between Glu233, water, and the glycosidic oxygen.

Altogether, we believe that these studies collectively demonstrate how advances in multiscale modelling for characterizing enzyme reactivity and dynamics have provided compelling insight into the nature of enzyme reactivity and catalysis. Multiscale modelling of enzymes should thus be regarded as a valuable complement to experimental research, towards a better understanding of enzyme evolution, catalytic reactivity and technological potential, and the rapid development of the field will undoubtedly enable its application to address increasingly complex questions about these still highly enigmatic systems.

## 7 Conclusions and future perspectives

Computational enzymology has evolved into a field capable of addressing enzymatic catalysis with a level of detail and realism that would have been difficult to imagine even a decade ago. The developments reviewed here, ranging from structure prediction, molecular simulations, and quantum chemistry to enhanced sampling techniques and ML, have collectively reshaped how enzyme mechanisms are explored. Instead of relying on static structural models or manually proposed mechanisms, contemporary approaches increasingly treat enzymes as dynamic molecular systems, whose reactivity occurs from the interaction between the electronic structure, conformational fluctuations, and the surrounding environment.

Scheme 1 summarises a typical workflow for studying enzyme reaction mechanisms computationally. The workflow begins with obtaining a reliable 3D enzyme structure and an enzyme–substrate complex, derived from experimental data or *in silico* modelling. Following model refinement to correct structural inconsistencies and assign protonation states, MD simulations are usually performed to equilibrate the system and sample conformational space. Representative snapshots of catalytically competent conformations are then selected to proceed for mechanistic studies using hybrid QM/MM approaches or ML potentials trained on quantum chemical data. The choice of method very much depends on what is desired and available, such as chemical accuracy, computational power, time constraints, or the availability of a suitable database. By repeating calculations across multiple conformations, researchers can analyse energy barrier variations to identify catalytically relevant interactions and to discriminate between competing mechanistic hypotheses. The workflow concludes with validation and refinement to ensure robust mechanistic insight.

A critical aspect of this workflow (Scheme 1) is the propagation of errors across modelling stages. Each step introduces its own sources of error, from structural modelling, limited conformational sampling during MD simulations and multi-PES calculations to the inherent approximations of hybrid QM/MM or ML methods. Because these steps are sequential, initial choices can influence the configurational space explored in subsequent steps, significantly impacting mechanistic conclusions. Quantifying these uncertainties is difficult, as errors rarely combine in a straightforward additive way. Instead, they may introduce systematic errors if key features, such as substrate pose or protonation states, are misrepresented.

Nevertheless, some errors can be estimated, such as statistical errors or uncertainties arising from methodological choices. For instance, errors associated with DFT-calculated barriers are typically on the order of 2–5 kcal mol^−1^, depending on the functional employed.^460–462^ QM/MM calculations introduce uncertainties, which can amount to just a few kcal mol^−1^, related to the definition of the QM region and embedding scheme,^268,463,464^ while free energy sampling errors, though sampling dependent, are *ca*. 1–2 kcal mol^−1^.^336,341,465,466^ In the case of enzymatic activation barriers, the uncertainty predicted by ML potentials can be a few kcal mol^−1^, but can become substantially larger, for example, when rare reactive configurations, particularly in the TS region, are underrepresented in the training set.^466–468^

In the end, comparing computed and experimental properties provides a measure of the propagated error. The calculated activation free energy is particularly sensitive to computational inaccuracies. In our experience, with well-balanced protocols where the final error is not dominated by a single source of uncertainty, systematic errors can lead to differences between computed and measured activation free energies within 3–5 kcal mol^−1^. Although these errors can seem large, as we address fine problems such as enzyme redesign or enzyme inhibition, we can still properly capture the thermodynamics of enzyme reaction mechanisms and fine changes in the mechanism, when controlled modifications are systematically introduced in the systems under study.

An important message from this review is that enzymatic catalysis is basically multiscale and dynamic. On the one hand, classical MD simulations provide access to substrate recognition, conformational flexibility, and long-range environmental effects. On the other hand, quantum mechanical methods are required to describe bond breaking and bond formation, while hybrid QM/MM approaches connect these regimes in a physically meaningful manner. Enhanced sampling techniques further extend this framework by enabling the quantitative characterization of rare, but catalytically decisive, events that are not accessible to conventional simulations. Together, these methods provide a sound picture of where chemical reactivity stands within the broader dynamical behaviour of enzymes.

From soluble enzymes to enzymes acting at complex interfaces, such as membrane associated catalysts, it is now evident that catalytic rates across a wide range of systems show ensemble behaviour rather than a single reactive structure. Small changes in active site geometry, hydration or residue positioning can convert into substantial variations in activation free energies, even when the overall chemical transformation remains unchanged.

Rate enhancement can be rationalised conceptually by the notion of electrostatic preorganization, but it is increasingly clear that this preorganization is dynamic and continuously modulated by protein motions, solvent rearrangements, and substrate binding modes. Therefore, capturing these effects requires explicit sampling of conformational and environmental degrees of freedom.

Together with methodological advances, the increasing availability of validated mechanistic and structural enzymology data has opened new potential for mechanism discovery. Annotated datasets such as the Mechanism and Catalytic Site Atlas and EzCatDB reveal that mechanistic similarity does not always follow evolutionary relationships, emphasising the existence of independent solutions to common catalytic challenges.

Based on all this knowledge, a new generation of computational tools has appeared, which automatically generates mechanistic hypotheses based on known catalytic patterns. Approaches such as EzMechanism can move beyond a single pathway rational by building networks of chemically reasonable catalytic routes that connect reactants to products. The emergence of multiple alternative pathways is a consequence of the limited set of functional groups typically involved in catalysis and the natural flexibility of enzymatic active sites. This interpretation is consistent with a rising body of experimental and computational evidence that indicates how enzymes can access distinct reaction routes depending on the local geometry, hydration and/or conformational state.

Knowledge-based approaches alone, however, are not sufficient to establish pathways that are both energetically and kinetically relevant, which means that physics-based methods remain crucial. Combining automated hypotheses with rigorous QM/MM validation represents a particularly promising path, because it allows the systematic exploration of large reactional spaces that would be difficult to study using a manual approach only.

Looking ahead, several developments are expected to shape the near future of computational enzymology. The tighter combination of knowledge-based tools with multiscale simulations will simplify the systematic generation and testing of mechanistic hypotheses. ML is expected to play an increasingly important role, by accelerating simulations, identifying meaningful CVs and organizing the huge data generated by modern computational calculations. This evolution highlights the critical relevance of FAIR principles (findable, accessible, interoperable and reusable). Indeed, communities that prioritized data curation decades ago (*e.g.*, structural biology community) are now collecting the greatest rewards from the AI revolution. In contrast, fields like biomolecular simulation still lack systematic maintenance. Establishing FAIR-compliant repositories would undoubtedly enhance the impact, reproducibility, and knowledge democratization, while fuelling further advances in artificial intelligence.^469^

Simultaneously, simulations that explicitly account for realistic environments, including membranes, crowded cellular contexts and heterogeneous interfaces, will become increasingly routine as computational resources and multiscale modelling strategies improve. These environments are active participants in catalysis, influencing substrate access, electrostatics, hydration, and conformational equilibria. Combining all this complexity will be essential for bridging the gap between computational mechanistic studies and realistic enzymatic function in both physiologically and technologically relevant conditions.

Despite all these advances, important challenges remain, *e.g.*, rigorous uncertainty quantification, transferability of MLIPs or standardisation of mechanistic data formats and require continued community effort. Continued progress will depend heavily on a closer combination of computation and experiment, not only for validation but also for the joint design of experiments that directly probe mechanistic hypotheses generated *in silico*.

As computational advances become more automated, more accurate, and more closely combined with experimental observables, they are expected to play an increasingly important role in enzyme engineering, inhibitor design, and the development of novel biocatalysts. In this evolving landscape, computation has rapidly shifted from an interpretative to a predictive tool, capable of identifying concealed catalytic motifs, guiding mutagenesis strategies, and selecting experimental efforts within diverse reactional spaces. Particularly exciting is the rational design of enzymes capable of synthesizing small complex molecules within (chemo)-enzymatic cascades. Furthermore, newly accessible applications include tailoring enzymes to modify biological therapeutics and upcycling or recycling of plastic waste by degrading synthetic polymers.^470^

In summary, computational enzymology is rapidly evolving towards an impressive area of knowledge in which mechanistic understanding, multiscale simulations and ML operate in synergy. While enzymes remain among the most complex molecular systems studied in chemistry and biochemistry, the approaches reviewed here have rapidly improved our ability to understand, predict, and ultimately control enzymatic reactivity, placing computer calculations at the centre of future advances in catalysis and biotechnology.

## Author contributions

All authors contributed to the literature review, writing and editing of the manuscript, and approved the final version. Rui P. P. Neves: conceptualization, investigation (equal), writing – original draft (equal), writing – review and editing, visualization. João T. S. Coimbra: conceptualization, investigation (equal), writing – original draft (equal), writing – review and editing, visualization. Pedro Paiva: conceptualization, investigation (equal), writing – original draft (equal), writing – review and editing. Umberto Raucci: investigation (equal), writing – original draft (equal), writing – review and editing, visualization. Sudip Das: investigation (equal), writing – original draft (equal), writing – review and editing. Ana R. Calixto: investigation (equal), writing – original draft (equal), writing – review and editing, visualization. António J. M. Ribeiro: investigation (equal), writing – original draft (equal), writing – review and editing, visualization. João P. M. Sousa: investigation (equal), writing – original draft (equal), writing – review and editing, visualization. Pedro Ferreira: investigation (equal), writing – original draft (equal), writing – review and editing. Enrico Trizio: investigation, writing – review and editing, visualization. Pedro A. Fernandes: writing – review and editing. Michele Parrinello: conceptualization, writing – review and editing, supervision. Maria J. Ramos: conceptualization, writing – original draft, writing – review and editing, supervision.

## Conflicts of interest

There are no conflicts to declare.

## Data Availability

No new data were generated or analysed in this review. All information was obtained from previously published sources cited in the manuscript.
